# Robust Distributed Collaborative Beamforming for Wireless Sensor Networks with Channel Estimation Impairments

**DOI:** 10.3390/s19051061

**Published:** 2019-03-02

**Authors:** Oussama Ben Smida, Slim Zaidi, Sofiène Affes, Shahrokh Valaee

**Affiliations:** 1 INRS-EMT, Université du Québec, Montreal, QC H5A 1K6, Canada; zaidi@emt.inrs.ca (S.Z.); affes@emt.inrs.ca (S.A.); 2 ECE Department, University of Toronto, Toronto, ON M5S 3G4, Canada; valaee@utoronto.ca

**Keywords:** collaborative beamforming (CB), distributed CB (DCB), robust DCB (RDCB), wireless sensor network (WSN), scattering, channel mismatch, implementation impairments, channel estimation errors, synchronization, localization, direction-of-arrival (DoA), scatterers

## Abstract

We propose a new collaborative beamforming (CB) solution robust (i.e., RCB) against major channel estimation impairments over dual-hop transmissions through a wireless sensor network (WSN) of *K* nodes. The source first sends its signal to the WSN. Then, each node forwards its received signal after multiplying it by a properly selected beamforming weight. The latter aims to minimize the received noise power while maintaining the desired power equal to unity. These weights depend on some channel state information (CSI) parameters. Hence, they have to be estimated locally at each node, thereby, resulting in channel estimation errors that could severely hinder CB performance. Exploiting an efficient asymptotic approximation at large *K*, we develop alternative RCB solutions that adapt to different implementation scenarios and wireless propagation environments ranging from monochromatic (i.e., scattering-free) to polychromatic (i.e., scattered) ones. Besides, in contrast to existing techniques, our new RCB solutions are distributed (i.e., DCB) in that they do not require any information exchange among nodes, thereby dramatically improving both WSN spectral and power efficiencies. Simulation results confirm that the proposed robust DCB (RDCB) techniques are much more robust in terms of achieved signal-to-noise ratio (SNR) against channel estimation errors than best representative CB benchmarks.

## 1. Introduction

Collaborative beamforming (CB) stands out today as a key technique that offers tremendous capacity, coverage, and power gains [1,2,3,4,5,6,7,8,9,10,11,12,13,14,15,16,17,18,19,20,21,22,23,24]. Using CB, *K* autonomous and independent sensor nodes relay the information from a desired source to a target destination through a two-hop communication link by estimating then transmitting weighted replicas of the desired signal in the first and second time slots, respectively. The beamforming weights are designed so as to optimize an objective function while satisfying some practical constraints. Due to its numerous merits, CB has gained the attention of the research community. Ref. [2] introduced the CB concept and analyzed its performance in wireless sensor networks (WSNs). Ref. [12] evaluated the CB’s beampattern characteristics while [13] designed a technique that narrows down its mainbeam and minimizes its sidelobe effect. Refs. [14,15] proposed CB solutions that improve WSN energy efficiency and reduce its nodes collaboration time while [17,18,19,20,21,22,23,24] extended the CB applicability range to scattered environments.

Nevertheless, CB’s implementation require all other beamforming nodes’s information such as their locations. This can entail an excessively large amount of information exchange among the nodes. The optimal beamforming weights may then be efficiently approximated by another set of weights that can be computed based solely on locally available information at each individual node, thereby creating a distributed CB (DCB) solution capable of relieving the nodes from the cumbersome information exchange procedure.

Despite its advantages, CB (or DCB) inevitably suffers in practice from channel estimation errors. Indeed, the beamforming weights often depend on and, hence, require the estimation of channel state information (CSI) parameters locally at each node. Unfortunately, such a process could result in several estimation errors that may cause severe channel mismatch and, hence, dramatically hinder the CB (or DCB) performance. To overcome this shortcoming, Refs. [25,26,27,28,29,30,31,32,33,34,35,36,37,38] developed new robust CB (RCB or RDCB) techniques against such estimation errors. These techniques could be roughly divided into two categories: worst-case and stochastic. The former are designed to handle the worst-case scenario when errors reach their maximum and, hence, can be extremely inefficient when actually these errors are in most real-world conditions subject to random perturbations and/or unbounded [38,39]. The latter are more robust since their design accounts for random errors. Nevertheless, they have some drawbacks of their own. Indeed, they rely very often on iterative greedy suboptimal search approaches that explore a daunting number of potential solutions. Unfortunately, WSN nodes find their extremely limited computing and power capabilities severely burdened and quickly exhausted or depleted. Besides, their robustness very often deteriorate drastically in the presence of large channel estimation errors and, hence, become unsuitable for hostile wireless environments. More importantly, almost all existing stochastic CB techniques suffer from another major drawback: the key fact that they do not offer distributed solutions. Indeed, the weights depend on other node information which are locally unavailable. Although robust to small errors, their implementation requires in real-world operating conditions huge information exchange among all nodes. The required overwhelming data overhead could starve to "death" the very limited computing and power capabilities of WSN nodes, very often found already exhausted and depleted (cf. above), and, if not enough, could dramatically degrade their spectrum efficiency. [32] proposed a stochastic CB solution that is both robust and distributed (i.e., RDCB) where the total transmission power of the relays is minimized while being subject to an outage probabilistic quality of service (QoS) constraint. Nevertheless, the RDCB in [32] assumes that estimation errors relatively are much smaller than the channel estimates and, hence, suffers from severe performance degradation in harsh environments that characterize real-world operating conditions.

We propose a new RDCB solution robust against major channel estimation impairments, namely phase synchronization, localization, direction-of-arrival (DoA), and/or channel scatterers/coefficients estimation errors over dual-hop transmissions from a source to a destination communicating through a WSN of *K* nodes. In the first time slot, the source sends its signal to the WSN while, in the second, each node forwards its received signal after multiplying it by a properly selected beamforming weights. The latter aims to minimize the received noise power while maintaining the desired power equal to unity. These weights depend a priori on some CSI parameters. Hence, they have to be estimated locally at each node, thereby resulting in channel estimation errors that could severely hinder the CB performance. Exploiting an efficient asymptotic approximation at large *K*, we develop alternative solutions that not only account for estimation errors, but also adapt to different implementation scenarios and wireless propagation environments ranging from monochromatic (i.e., scattering-free) to polychromatic (i.e., scattered) ones. Besides, in contrast to existing techniques, our new RCB solutions are also distributed (i.e., RDCB) in that they do not require any information exchange among nodes, thereby dramatically improving the WSN spectral and power efficiencies. Simulations results confirm that the proposed RDCB techniques are much more robust in terms of achieved signal-to-noise ratio (SNR) against channel estimation errors than the nominal optimal CB solution (i.e., optimized without being aware of and, hence, accounting for impairments) and RDCB in [32] benchmarks, yet at much lower complexity, power cost, and overhead, making them suitable for WSN deployment in the harsh environments that characterize operation in real-world conditions.

The paper is organized as follows. Section 2 describes the dual-hop communication system model. The proposed monochromatic RDCB (M-RDCB) and polychromatic RDCB (P-RDCB) techniques are derived in Section 3. Section 4 analyzes theoretically the performance gains that the new M-RDCB and P-RDCB solutions could potentially achieve by integrating robustness in their designs in monochromatic and polychromatic environments. Simulation results are discussed in Section 5 and Section 6 draws out concluding remarks.

Notation: Uppercase and lowercase bold letters denote matrices and column vectors, respectively. ${\left[.\right]}_{il}$ and ${\left[.\right]}_{i}$ are the ${\left(i,l\right)}^{th}$ entry of a matrix and $i^{th}$ entry of a vector, respectively. The operators ${\left(.\right)}^{*}$, ${\left(.\right)}^{T}$, and ${\left(.\right)}^{H}$ denote the complex conjugate, the transpose, and the complex conjugate transpose or Hermitian, respectively. ||.|| is the 2-norm of a vector and |.| is the absolute value. The operator ⊙ is the element-wise product. E{.} stands for the statistical expectation and $J_{1}\left(.\right)$ is the first-order Bessel function of the first kind.

## 2. System Model

**Figure 1:**
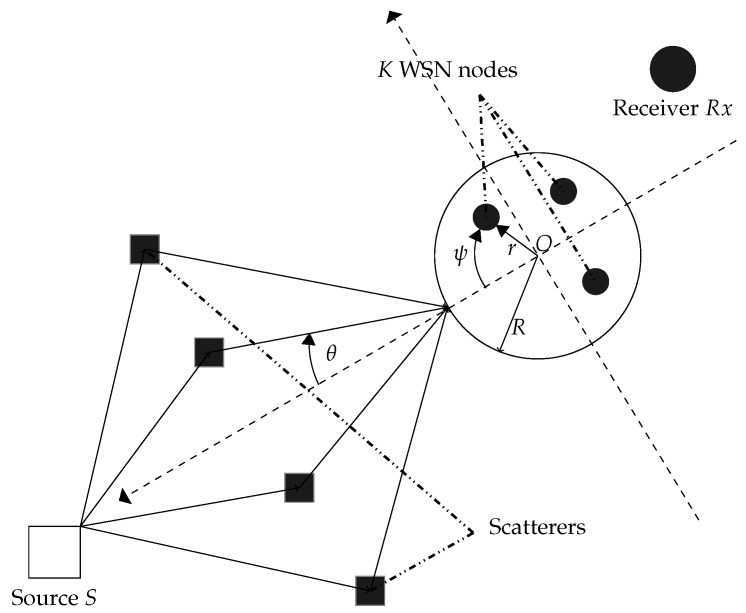
System model.

As illustrated in Figure 1, the system of our interest consists of a wireless sensor network (WSN) comprised of *K* nodes each of which is equipped with a single isotropic antenna and uniformly and independently distributed on D(O,R), the disc with center at *O* and radius *R*, a receiver Rx, and a source *S* both located in the same plane containing D(O,R). We assume that there is no direct link from the source to the receiver due to high pathloss attenuation [2,15,17,18,19,20,21]. Moreover, let $\left(A_{s},\phi_{s}\right)$ denote the source’s polar coordinates and *s* its narrow-band. In this paper, we assume that the signal bandwith’s reciprocal is large with respect to the time delays of all rays. For this reason, the time notion is ignored when denoting the source signal. unit power signal. Without any loss of generality, *S* is assumed to be at $\phi_{s}=0$. Let $\left(r_{k},\psi_{k}\right)$, ${\left[g\right]}_{k}$, and ${\left[f\right]}_{k}$ denote the *k*-th node’s polar coordinates, backward, and forward channel, respectively. ${\left[f\right]}_{k}$ is assumed to be a zero-mean unit-variance circular Gaussian random variable. Since the WSN nodes are independent and completely autonomous, we consider here that the *k*-th WSN is only aware of its coordinates and both its backward and forward channels while being obvious to those of all other nodes in the network. A dual-hop communication is established from the source *S* to the receiver Rx. In the first time slot, the source sends its signal *s* to the WSN. Let y denote the received signal vector at the sensor nodes given by

(1) y=gs+v,

where $g≜{\left[{\left[g\right]}_{1}\ldots {\left[g\right]}_{K}\right]}^{T}$ and v is the nodes’ noise vector. In the second time slot, the *k*-th node multiplies its received signal with the complex conjugate of the beamforming weight $w_{k}$ and forwards the resulting signal to the receiver. It follows from (1) that the received signal at *O* is

(2) $$\begin{array}{ccc} r & = & f^{T}\left(w^{*}⊙y\right)+n=w^{H}\left(f⊙y\right)+n\\ & = & w^{H}\left(f⊙gs+f⊙v\right)+n\\ & = & sw^{H}h+w^{H}\left(f⊙v\right)+n, \end{array}$$

where $w≜[w_{1}\ldots w_{K}]$ is the beamforming vector, h≜f⊙g, $f≜{\left[{\left[f\right]}_{1}\ldots {\left[f\right]}_{K}\right]}^{T}$, and *n* is the receiver noise. Let $P_{w,s}$ and $P_{w,n}$ denote the received power from the source, and the aggregate noise power due to the thermal noise at the receiver and the forwarded noises from the terminals, respectively. It holds from (2) that

(3) $$\begin{array}{ccc} P_{w}\left(\phi_{s}\right) & = & {\left|w^{H}h\right|}^{2}, \end{array}$$

(4) $$\begin{array}{ccc} P_{w,n} & = & \sigma_{v}^{2}w^{H}\Lambda w+\sigma_{n}^{2}, \end{array}$$

where $\Lambda ≜\mathrm{diag}\{|{\left[f\right]}_{1}{|}^{2}\ldots |{\left[f\right]}_{K}{|}^{2}\}$. Although several approaches can be adopted to properly design the beamforming weights [35], we are only concerned in this paper with minimizing the aggregate noise power while maintaining the beamforming response $w^{H}h$, and hence, the power received from the source equal to unity. The latter is nothing else but the well-known minimum variance distortionless response (MVDR) beamformer [40,41] with a relaxed distortionless response constraint. Mathematically speaking, we have to solve the following optimization problem:

(5) $$w_{\mathrm{opt}}=\arg\min P_{w,n}\;s.t.\;P_{w}\left(\phi_{s}\right)=1,$$

where $w_{\mathrm{opt}}$ denotes the ideal optimal beamforming vector. The optimization problem in (5) can be rewritten as

(6) $$w_{\mathrm{opt}}=\arg\min w^{H}\Lambda w\;s.t.\;{\left|w^{H}h\right|}^{2}=1,$$

or, equivalently as

(7) $$w_{\mathrm{opt}}=\arg\max\frac{w^{H}hh^{H}w}{w^{H}\Lambda w}\;\;s.t.\;\;{\left|w^{H}h\right|}^{2}=1.$$

The solution of the above convex optimization problem can be expressed as

(8) $$w_{\mathrm{opt}}=\frac{\Lambda^{-1}h}{\left|h^{H}\Lambda^{-1}h\right|},$$

and, hence, the *k*-th node’s weight is given by

(9) $${\left[w_{\mathrm{opt}}\right]}_{k}=\frac{{\left[h\right]}_{k}}{{|\left[f\right]}_{k}{||\left[g\right]}_{k}|}.$$

It follows from (9) that in order to implement $w_{\mathrm{opt}}$, the *k*-th node must estimate both its backward ${\left[f\right]}_{k}$ and forward channels ${\left[g\right]}_{k}$. Unfortunately, in practice, such a process results in channel estimation errors which may hinder the beamforming performance. As such, $w_{\mathrm{opt}}$ is only valid in ideal conditions where implementation impairments do not exist. In real-world conditions, $w_{\mathrm{opt}}$ is substituted by

(10) $${\tilde{w}}_{\mathrm{opt}}=\frac{{\tilde{\Lambda }}^{-1}\tilde{h}}{\left|{\tilde{h}}^{H}{\tilde{\Lambda }}^{-1}\tilde{h}\right|},$$

where ${\left[\tilde{f}\right]}_{k}$ and ${\left[\tilde{g}\right]}_{k}$ are the *k*-th estimates of the backward and forward channels, respectively. Another drawback of the nominal optimal CB solution (as referred to in the remainder of the paper) ${\tilde{w}}_{\mathrm{opt}}$ (i.e., optimized without being aware of and, hence, accounting for channel parameter estimation impairments), which must be emphasized herein, is that the *k*-th node must be aware of the channel estimates of all other nodes in the WSN. To this end, each node must broadcast its channel information through the network and, hence, ${\tilde{w}}_{\mathrm{opt}}$’s implementation requires a huge overhead. The latter might not only cause the depletion of the WSN nodes scarce energy resources and the deterioration of their spectral efficiency, but also from severely amplified performance losses due to the cumulatively increasing impact of CSI feedback errors. In what follows, we propose new RDCB techniques robust against channel parameter estimation errors both in monochromatic and polychromatic environments. We focus in this work on phase synchronization, localization, DoA, and/or channel scatterers/coefficients estimation errors.

## 3. Proposed Robust Distributed Collaborative Beamforming (RDCB) Techniques

In order to overcome channel estimation impairments, one should start by taking an in-depth look into the backward channel structure. The latter falls under two main categories: (i) single-ray (i.e., monochromatic) channels that ignore the scattering phenomenon to assume only a unique line-of-sight ray; and (ii) multi-ray (i.e., polychromatic) ones that account for the scattering present in most real-world environments. As far as the forward channel is concerned, we make no particular a priori assumption on its structure (i.e., whether it is monochromatic or polychromatic). And so we assume that its estimate includes an additive channel estimation error term.

### 3.1. Monochromatic (i.e., Scattering-Free) Environments

In such environments, ${\left[g\right]}_{k}$ can be expressed as

(11) $${\left[g\right]}_{k}=e^{-j\varrho_{k}},$$

where $\varrho_{k}=\frac{2\pi }{\lambda }r_{k}\cos\left(\phi_{s}-\psi_{k}\right)$ is the *k*-th node’s initial phase. Hence, the corresponding beamforming weight can be estimated from a pilot signal received at each node from the source *S*, assumed in this case to be far from the nodes (i.e., $A_{s}\gg R$) along two options: (1) either each node estimates the initial phase $\varrho_{k}$; or (2) it estimates both the direction-of-arrival (DoA) $\phi_{s}$ and the coordinates $\left(r_{k},\psi_{k}\right)$. The first option requires the implementation of phase synchronization techniques [42,43] while the second relies both on DoA estimation and localization algorithms [44,45]. Nevertheless, both options incur estimation errors of different nature that hinder the accuracy of ${\left[g\right]}_{k}$ and, hence, the performance of CB.

#### 3.1.1. Implementation Option 1 (Phase Synchronization)

This implementation option results in a phase jitter due to synchronization and phase offset estimation errors among nodes. Therefore, the *k*-th node’s backward channel estimate ${\left[\tilde{g}\right]}_{k}$ is given as

(12) $${\left[\tilde{g}\right]}_{k}=e^{-j\varrho_{k}}\Delta_{g_{k}},$$

where $\Delta_{g_{k}}=e^{-j\delta_{k}}$ and $\delta_{k}$ is the *k*-th node’s phase jitter that depends on its local oscillator characteristics. We will show later that ${\tilde{w}}_{\mathrm{opt}}$’s performance deteriorates as $\delta_{k}$ increases due to channel mismatch (i.e., ${\left[\tilde{g}\right]}_{k}\neq {\left[g\right]}_{k}$) it causes. To overcome this challenging issue, we propose in this paper to anticipate the inevitable phase jitter by accounting for its impact in the CB design. Actually, one could modify the optimization problem in (7) as

(13) $$w_{{\mathrm{MR}}_{1}}=\arg\max\frac{w^{H}\tilde{h}{\tilde{h}}^{H}w}{w^{H}\Lambda w}\;\;s.t.\;\;{\left|w^{H}h\right|}^{2}=1,$$

where ${\left[\tilde{h}\right]}_{k}={\left[\tilde{f}\right]}_{k}{\left[\tilde{g}\right]}_{k}$, ${\left[\tilde{f}\right]}_{k}={\left[f\right]}_{k}+\Delta_{f_{k}}$, and $\Delta_{f_{k}}$ is the error incurred when estimating the *k*-th node’s backward channel without a priori knowledge of its structure (i.e., whether monochromatic or polychromatic) using a training sequence sent from the receiver. The robust proposed beamforming vector is then given by

(14) $$w_{{\mathrm{MR}}_{1}}=\frac{{\tilde{\Lambda }}^{-1}\tilde{h}}{|{\tilde{h}}^{H}{\tilde{\Lambda }}^{-1}h|}.$$

As can be observed from (14), $w_{{\mathrm{MR}}_{1}}$ depends on both the actual and estimated channel values. Because nodes are of course unaware of the actual channel, we need to substitute $\left|{\tilde{h}}^{H}{\tilde{\Lambda }}^{-1}h\right|$ by an equivalent quantity that depends only on known parameters. To this end, we propose to investigate the asymptotic expression for this term at large *K*. It follows from the definitions of $\tilde{h}$, $\tilde{\Lambda }$, and h that

(15) $$\begin{array}{ccc} {\left({\tilde{h}}^{H}{\tilde{\Lambda }}^{-1}h\right)}^{H}\left({\tilde{h}}^{H}{\tilde{\Lambda }}^{-1}h\right) & = & {\left(\sum_{k=1}^{K}\frac{{\left[\tilde{g}\right]}_{k}^{H}{\left[\tilde{f}\right]}_{k}^{H}{\left[f\right]}_{k}{\left[g\right]}_{k}}{{|\left[f\right]}_{k}{|}^{2}}\;\right)}^{\;\;H}\;\;\;\;\left(\sum_{p=1}^{K}\frac{{\left[\tilde{g}\right]}_{p}^{H}{\left[\tilde{f}\right]}_{p}^{H}{\left[f\right]}_{p}{\left[g\right]}_{p}}{{|\left[f\right]}_{p}{|}^{2}}\right)\\ & = & K+K\left(K-1\right)\frac{\sum_{k=1}^{K}\;\Delta_{g_{k}}}{K}\frac{\sum_{p=1,p\neq k}^{K}\;\Delta_{g_{p}}^{H}}{K-1}+K\frac{\sum_{k=1}^{K}\Delta_{f_{k}}^{2}}{K}\\ & + & K\left(K-1\right)\frac{\sum_{k=1}^{K}\;\frac{\Delta_{g_{k}}{\left[f\right]}_{k}^{H}\Delta_{f_{k}}}{{|\left[f\right]}_{k}{|}^{2}}}{K}\frac{\sum_{p=1,p\neq k}^{K}\;\frac{\Delta_{g_{p}}^{H}{\left[f\right]}_{p}\Delta_{f_{p}}}{{|\left[f\right]}_{p}{|}^{2}}}{K-1}. \end{array}$$

By resorting to the law of large numbers, we have for large *K* that $\frac{1}{K}\sum_{k=1}^{K}\;\Delta_{g_{k}}≃E\left\{\Delta_{g_{k}}\right\}$, $\frac{1}{K-1}\sum_{p=1,p\neq k}^{K}\;\Delta_{g_{p}}^{H}≃E\left\{\Delta_{g_{p}}^{H}\right\}$, $\frac{1}{K}\sum_{k=1}^{K}\Delta_{f_{k}}^{2}≃E\left\{\Delta_{f_{k}}^{2}\right\}$, $\frac{1}{K}\sum_{k=1}^{K}\;\frac{\Delta_{g_{k}}{\left[f\right]}_{k}^{H}\Delta_{f_{k}}}{{\left[f\right]}_{k}^{2}}≃E\left\{\Delta_{g_{k}}\right\}E\left\{\frac{{\left[f\right]}_{k}^{H}}{{|\left[f\right]}_{k}{|}^{2}}\right\}E\left\{\Delta_{f_{k}}\right\}$, and $\frac{1}{K-1}\sum_{p=1,p\neq k}^{K}\;\frac{\Delta_{g_{p}}^{H}{\left[f\right]}_{p}\Delta_{f_{p}}}{{|\left[f\right]}_{p}{|}^{2}}≃E\left\{\Delta_{g_{p}}^{H}\right\}E\left\{\frac{{\left[f\right]}_{p}}{{|\left[f\right]}_{p}{|}^{2}}\right\}E\left\{\Delta_{f_{p}}\right\}$. Assuming that $\delta_{k}$ and $\Delta_{f_{k}}$ are independent and uniformly distributed over $\left[-\sqrt{3}\sigma_{g},\sqrt{3}\sigma_{g}\right]$ and $\left[-\sqrt{3}\sigma_{f},\sqrt{3}\sigma_{f}\right]$, respectively, one could obtain for large *K*

(16) $$\begin{array}{ccc} \left|{\tilde{h}}^{H}{\tilde{\Lambda }}^{-1}h\right| & ≃ & \sqrt{K+K\left(K-1\right)E^{2}\left\{\Delta_{g_{k}}\right\}+KE\left\{\Delta_{f_{k}}^{2}\right\}}\\ & ≃ & \;\;\sqrt{K\left(1+\sigma_{f}^{2}\right)+K\left(K-1\right)\frac{\sin^{2}\left(\sqrt{3}\sigma_{g}\right)}{3\sigma_{g}^{2}}}, \end{array}$$

where $\sigma_{g}$ and $\sigma_{f}$ are the variances of $\delta_{k}$ and $\Delta_{f_{k}}$, respectively. As the number of nodes in WSNs is typically large, we can substitute (16) in (14) to obtain

(17) $$w_{{\mathrm{MR}}_{1}}≃\frac{{\tilde{\Lambda }}^{-1}\tilde{h}}{\sqrt{K\left(1+\sigma_{f}^{2}\right)+K\left(K-1\right)\frac{\sin^{2}\left(\sqrt{3}\sigma_{g}\right)}{3\sigma_{g}^{2}}}}.$$

A straightforward inspection of (17) reveals that ${\left[w_{{\mathrm{MR}}_{1}}\right]}_{k}$ is exclusively dependent on ${\left[\tilde{f}\right]}_{k}$, ${\left[\tilde{g}\right]}_{k}$, $\sigma_{g}$, and $\sigma_{f}$. The first and second are locally estimated by the *k*-th node while ${\left[\tilde{g}\right]}_{k}$ and $\sigma_{g}$ depend on its local oscillator characteristics and the adopted phase synchronization technique and, hence, could be stored in its local memory before WSN deployment. Furthermore, ${\left[w_{{\mathrm{MR}}_{1}}\right]}_{k}$ is independent of the forward and backward channels of all other nodes. This is an important DCB feature since it avoids any information exchange among WSN nodes thereby saving their scarce energy resources and improving the WSN spectral efficiency. It is worth noting in the absence of backward channel phase estimation errors (i.e., $\sigma_{g}=0$) and forward channel estimation errors (i.e., $\sigma_{f}=0$) that $w_{{\mathrm{MR}}_{1}}$ reduces to the nominal M-DCB solution given in [18] by

(18) $$w_{M}=\frac{\Lambda^{-1}h}{K},$$

thereby underlining unambiguously both the challenges and the merits of implementing DCB robustness to channel parameter estimation errors.

#### 3.1.2. Implementation Option 2 (Localization and direction of arrival (DoA) Estimation)

With option 2, each node must perform both self-localization and DoA estimation with, once again, some inevitable estimation errors that hinder the channel information accuracy. In such a case, the estimated backward channel can be written as

(19) $$\begin{array}{ccc} {\left[\tilde{g}\right]}_{k} & = & e^{-j\frac{2\pi }{\lambda }\left(r_{k}+\delta_{r_{k}}\right)\cos\left(\psi_{k}+\delta_{\psi_{k}}\right)}, \end{array}$$

where $\delta_{r_{k}}$ is the error on the radial coordinate $r_{k}$ and $\delta_{\psi_{k}}$ is the combined error on the angle coordinate $\psi_{k}$ and $\phi_{s}$ ($\phi_{s}$ = 0). Adopting similar steps as in Section 3.1.1, one can prove that the proposed RDCB can be expressed in this scenario as

(20) $$w_{{\mathrm{MR}}_{2}}≃\frac{{\tilde{\Lambda }}^{-1}\tilde{h}}{\sqrt{\;K\left(1+\sigma_{f}^{2}\right)+K\left(K-1\right)E{\left\{e^{j\frac{2\pi }{\lambda }\left(\nu_{k}-2R\mu_{k}\sin\left(\frac{\delta_{\psi_{k}}}{2}\right)\right)}\right\}}^{2}}},$$

where expectation is taken over $\nu_{k}$, $\mu_{k}$, $\delta_{\psi_{k}}$, $\nu_{k}=\delta_{r_{k}}\cos\left(\psi_{k}+\delta_{\psi_{k}}\right)$, and $\mu_{k}=\frac{r_{k}}{R}\sin\left(\psi_{k}+\frac{\delta_{\psi_{k}}}{2}\right)$. As could be observed from (17), each node is able to compute its own weight using only its local information, thereby avoiding any information exchange that may dramatically deteriorate the WSN power and spectral efficiencies. However, every node needs to compute the expectation in the right hand sight (RHS) of (17), thereby burdening the proposed beamformer’s implementation complexity. In what follows, we prove owing to the adoption of a mild assumption that its possible to derive this expectation term in (20) in closed form. Assuming that $\nu_{k}$ and $\mu_{k}$ are statistically independent, we have $E\left\{e^{j\frac{2\pi }{\lambda }\left(\nu_{k}-2R\mu_{k}\sin\left(\frac{\delta_{\psi_{k}}}{2}\right)\right)}\right\}=E_{\nu_{k}}\left\{e^{j\frac{2\pi }{\lambda }\nu_{k}}\right\}E_{\mu_{k},\delta_{\psi_{k}}}\left\{e^{-4j\pi R\mu_{k}\sin\left(\frac{\phi -\delta_{\psi_{k}}}{2}\right)}\right\}$. The pdf of $\nu_{k}$ can be determined as

(21) $$\begin{array}{ccc} f_{\nu_{k}}\left(\nu \right) & = & \frac{1}{2\pi \sqrt{3}\sigma_{r}}\left[\int_{\nu }^{\sqrt{3}\sigma_{r}}\frac{1}{\sqrt{\delta_{r}^{2}-\nu^{2}}}d\delta_{r}+\int_{-\sqrt{3}\sigma_{r}}^{-\nu }\frac{1}{\sqrt{\delta_{r}^{2}-\nu^{2}}}d\delta_{r}\right],\\ & = & \frac{1}{\pi \sqrt{3}\sigma_{r}}\left[\ln\left(1+\sqrt{1-\frac{\nu^{2}}{3\sigma_{r}^{2}}}\right)-\ln\left(\frac{\left|\nu \right|}{\sqrt{3}\sigma_{r}}\right)\right]\;\;\;\mathrm{with}\;\;\;\left|\nu \right|\leq \sqrt{3}\sigma_{r}. \end{array}$$

Therefore, its average is determined by

(22) $$\begin{array}{ccc} E_{\nu_{k}}\left\{e^{j\frac{2\pi }{\lambda }\nu_{k}}\right\} & = & \int_{-\sqrt{3}\sigma_{r}}^{\sqrt{3}\sigma_{r}}\frac{1}{\pi \sqrt{3}\sigma_{r}}e^{j\frac{2\pi }{\lambda }\nu_{k}}\left[\ln\left(1+\sqrt{1-\frac{\nu^{2}}{3\sigma_{r}^{2}}}\right)-\ln\left(\frac{\left|\nu \right|}{\sqrt{3}\sigma_{r}}\right)\right]d\nu .\\ & = & \frac{2}{\pi }\int_{0}^{1}\cos\left(\frac{2\pi }{\lambda }\sqrt{3}\sigma_{r}t\right)\ln\left(\frac{1+\sqrt{1-t^{2}}}{t}\right)dt\\ & = & {}_{1}F_{2}\left(0.5;1,1.5;-3{\left(\beta \left(\frac{\pi }{3}\right)\frac{\sigma_{r}}{2R}\right)}^{2}\right)\\ & = & \xi_{r}\left(\frac{\pi }{3}\right), \end{array}$$

where $\beta \left(\phi \right)=\frac{4\pi R}{\lambda }\sin\left(\frac{\phi }{2}\right)$. Please note in the second line that we resort to the variable change $t=\frac{\left|\nu \right|}{\sqrt{3}\sigma_{r}}$. We also remove the imaginary part of the equation as it is a sinus function which is odd and, hence, its integral over a zero-centered interval is null. Besides, we have

(23) $$\begin{array}{ccc} E_{\mu_{k},\delta_{\psi_{k}}}\left\{e^{-4j\pi R\mu_{k}\sin\left(\frac{\phi -\delta_{\psi_{k}}}{2}\right)}\right\} & = & E_{\delta_{\psi_{k}}}\left\{\sum_{p=0}^{+\infty }\frac{{\left(4\pi R\sin\left(\frac{-\delta_{\psi_{k}}}{2}\right)\right)}^{p}}{p!}{\left(-j\right)}^{p}E\left(\mu_{k}^{p}\right)\right\}\\ & = & E_{\delta_{\psi_{k}}}\left\{\frac{2J_{1}\left(4\pi R\sin\left(\frac{\delta_{\psi_{k}}}{2}\right)\right)}{4\pi R\sin\left(\frac{\delta_{\psi_{k}}}{2}\right)}\right\}\\ & ≃ & {\;}_{1}F_{2}\left(0.5;1.5,2;-3{\left(\pi \frac{R\sigma_{\psi }}{\lambda }\right)}^{2}\right)\\ & = & \xi_{\psi }\left(0\right). \end{array}$$

Injecting (22) and (23) in (20) yields (24)

(24) $$w_{{\mathrm{MR}}_{2}}≃\frac{{\tilde{\Lambda }}^{-1}\tilde{h}}{\sqrt{\;K\left(1+\sigma_{f}^{2}\right)\;+\;K{\left(K-1\right)}_{1}F_{2}{\left(\;0.5;1,1.5;-3{\left(\pi \frac{\sigma_{r}}{\lambda }\right)}^{2}\right)}^{2}\;\;\;{}_{1}F_{2}{\left(\;0.5;1.5,2;-3{\left(\pi \frac{R\sigma_{\psi }}{\lambda }\right)}^{2}\right)}^{2}}}.$$

in which $w_{{\mathrm{MR}}_{2}}$ depends only on the coefficients of own estimated channels, $\sigma_{r}$, $\sigma_{\psi }$, and $\sigma_{f}$. Since each terminal can locally estimate its own channel, the proposed M-RDCB solution does not incur any noticeable overhead, computation, or power costs. Whereas $\sigma_{r}$, $\sigma_{\psi }$, and $\sigma_{f}$ can be easily broadcast over the WSN at very negligible increase in such three cost items. It is also worth noting in absence of localization and DoA estimation errors (i.e., $\sigma_{r}=\sigma_{\psi }=0$) and forward channel estimation errors (i.e., $\sigma_{f}=0$) that the proposed M-RDCB, $w_{{\mathrm{MR}}_{2}}$, reduces once again to the nominal M-DCB solution $w_{M}$ given in Equation (18) above.

### 3.2. Polychromatic Environments

We assume here that the source is scattered by a given number of scatterers located in the same plane containing D(O,R). These scatterers generate out of the transmit signal *L* rays or “spatial chromatics” (with reference to their angular distribution) that form a polychromatic propagation channel. The *l*-th ray or chromatic is characterized by its angle deviation $\theta_{l}$ from the source direction $\phi_{s}$ and its complex amplitude $\alpha_{l}$. In such a case, the backward channel of the *k*-th node is given by

(25) $${\left[g\right]}_{k}=\sum_{l=1}^{L}\alpha_{l}e^{-j\frac{2\pi }{\lambda }r_{k}\cos\left(\phi_{s}+\theta_{l}-\psi_{k}\right)}.$$

It is noteworthy that (25) reduces to (11) when there is no scattering (i.e., $\theta_{l}=0$ and $\alpha_{l}=1/L$). It follows from (25) that each node must estimate in polychromatic environments its polar coordinates $\left(r_{k},\psi_{k}\right)$ and the *l*-th ray’s DoA $\phi_{s}+\theta_{l}$ and its amplitude $\alpha_{l}$. This would often result in errors which may cause a channel mismatch, thereby hindering the proposed beamforming performance. The backward channel estimate of the *k*-th node is then given by

(26) $${\left[\tilde{g}\right]}_{k}=\sum_{l=1}^{L}\left(\alpha_{l}+\delta_{\alpha_{l}}\right)e^{-j\frac{2\pi }{\lambda }\left(r_{k}+\delta_{r_{k}}\right)\cos\left(\theta_{l}-\psi_{k}+\delta_{kl}\right)},$$

where $\delta_{\alpha_{l}}$ and $\delta_{kl}$ are the errors on $\alpha_{l}$ and the combined phase $\left(\theta_{l}-\psi_{k}\right)$, respectively. It follows then from (26) that

(27) $$\begin{array}{c} {\tilde{h}}^{H}{\tilde{\Lambda }}^{-1}h=\sum_{l=1}^{L}\sum_{m=1}^{L}\sum_{k=1}^{K}{\tilde{\alpha }}_{l}^{*}\alpha_{e}^{j\beta \left(\theta_{l}-\theta_{m}+\delta_{kl}\right)\kappa_{klm}}e^{j\frac{2\pi }{\lambda }\vartheta_{kl}}+\sum_{k=1}^{K}\frac{{\left[\tilde{g}\right]}_{k}^{H}{\left[g\right]}_{k}{\left[f\right]}_{k}\Delta_{f_{k}}^{H}}{{|\left[f\right]}_{k}{|}^{2}}, \end{array}$$

where $\kappa_{klm}=r_{k}\sin\left(\psi_{k}-\frac{\theta_{l}+\theta_{m}+\delta_{kl}}{2}\right)$ and $\vartheta_{kl}=\delta r_{k}\cos\left(\psi_{k}-\theta_{l}-\delta_{kl}\right)$. Exploiting the law of large numbers and assuming that $\kappa_{klm}$ and $\vartheta_{kl}$ are statistically independent, we obtain at large *K*

(28) $$\begin{array}{ccc} |{\tilde{h}}^{H}{\tilde{\Lambda }}^{-1}{h|}^{2} & ≃ & K(\sum_{l,m,n,q=1}^{L}{\tilde{\alpha }}_{l}{\tilde{\alpha }}_{m}^{H}{\tilde{\alpha }}_{n}^{H}{\tilde{\alpha }}_{q}\left(\xi_{r}\left(\rho_{n,l}\right)\chi_{\psi }\left(\theta ,0\right)+\left(K-1\right)\xi_{r}\left(\frac{\pi }{3}\right)\tau_{\psi }\left(\theta ,0\right)\right)\\ & + & \sum_{\underset{m=q}{l,m,n=1}}^{L}{\tilde{\alpha }}_{l}{\tilde{\alpha }}_{n}^{H}\sigma_{\alpha }^{2}\left(\xi_{r}\left(\rho_{n,l}\right)\chi_{\psi }\left(\theta ,0\right)+\left(K-1\right)\xi_{r}\left(\frac{\pi }{3}\right)\tau_{\psi }\left(\theta ,0\right)\right)), \end{array}$$

where $\sigma_{\alpha }^{2}$ is the variance of $\delta_{\alpha }$ and $\chi_{\psi }\left(\theta ,\phi \right)$ is determined as follows

(29) $$\begin{array}{c} \chi_{\psi }\left(\theta ,\phi \right)=\\ E_{\delta_{\psi }}\left(\frac{2J_{1}\left(\sqrt{\beta {\left(\rho_{l,m}-\phi +\delta_{\psi_{k}}\;\right)}^{2}+\beta {\left(\rho_{n,q}-\phi +\delta_{\psi_{k}}\right)}^{2}+2\beta \left(\rho_{l,m}-\phi +\delta_{\psi_{k}}\right)\beta \left(\rho_{n,q}-\phi +\delta_{\psi_{k}}\right)\cos\left(\frac{\rho_{l,n}+\rho_{m,q}}{2}\right)}\right)}{\sqrt{\beta {\left(\rho_{l,m}-\phi +\delta_{\psi_{k}}\right)}^{2}+\beta {\left(\rho_{n,q}-\phi +\delta_{\psi_{k}}\right)}^{2}+2\beta \left(\rho_{l,m}-\phi +\delta_{\psi_{k}}\right)\beta \left(\rho_{n,q}-\phi +\delta_{\psi_{k}}\right)\cos\left(\frac{\rho_{l,n}+\rho_{m,q}}{2}\right)}}\right), \end{array}$$

where $\theta =\{\theta_{l},\theta_{m},\theta_{n},\theta_{q}\}$, $\rho_{l,m}=\theta_{l}-\theta_{m}$, and

(30) $$\tau_{\psi }\left(\theta ,\phi \right)=E_{\delta_{\psi }}\;\;\left(\;\frac{4J_{1}\left(\beta \left(\rho_{l,m}-\phi +\delta_{\psi_{k}}\right)\right)J_{1}\left(\beta \left(\rho_{n,q}-\phi +\delta_{\psi_{k}}\right)\right)}{\beta \left(\rho_{l,m}-\phi +\delta_{\psi_{k}}\right)\beta \left(\rho_{n,q}-\phi +\delta_{\psi_{k}}\right)}\;\right).$$

Injecting (28) in (14) yields the following new P-RDCB:

(31) $$\begin{array}{ccc} w_{\mathrm{PR}} & = & \frac{{\tilde{\Lambda }}^{-1}\tilde{h}}{K^{2}}(\sum_{l,m,n,q=1}^{L}{\tilde{\alpha }}_{l}{\tilde{\alpha }}_{m}^{H}{\tilde{\alpha }}_{n}^{H}{\tilde{\alpha }}_{q}\left(\xi_{r}\left(\rho_{n,l}\right)\chi_{\psi }\left(\theta ,0\right)+\left(K-1\right)\xi_{r}\left(\frac{\pi }{3}\right)\tau_{\psi }\left(\theta ,0\right)\right)\\ & + & \sum_{\underset{m=q}{l,m,n=1}}^{L}{\tilde{\alpha }}_{l}{\tilde{\alpha }}_{n}^{H}\sigma_{\alpha }^{2}\left(\xi_{r}\left(\rho_{n,l}\right)\chi_{\psi }\left(\theta ,0\right)+\left(K-1\right)\xi_{r}\left(\frac{\pi }{3}\right)\tau_{\psi }\left(\theta ,0\right)\right){)}^{-\frac{1}{2}}. \end{array}$$

Obviously, we observe from (31) that the *k*-th node requires knowledge of both $\chi_{\psi }\left(\theta ,0\right)$ and $\tau_{\psi }\left(\theta ,0\right)$ to be able to derive its corresponding weight ${\left[w_{\mathrm{PR}}\right]}_{k}$. However, to avoid the costly calculations of the integrals in (29) and (30), we derive in the sequel their expressions in closed form.

Let us first focus on $\chi_{\psi }\left(\theta ,0\right)$. Assuming that $\delta_{kl}$ is sufficiently small to satisfy $\sin\left(\delta_{kl}\right)≃\delta_{kl}$, $\chi_{\psi }\left(\theta ,0\right)$ could be rewritten as

(32) $$\begin{array}{c} \chi_{\psi }\left(\theta ,0\right)≃E_{\delta_{k,l},\delta_{k,n}}\left(\frac{2J_{1}\left(\beta \left(\pi \right)\sqrt{Z+c}\right)}{\beta \left(\pi \right)\sqrt{Z+c}}\right), \end{array}$$

where $Z=a\delta_{k,l}+b\delta_{kn}$ is a random variable whose pdf is

(33) $$\begin{array}{c} f_{Z}\left(z\right)=\left\{\begin{array}{c} 0\;\;\;\;\;\;\;\;\;\;\;\;\;\;\;\;\;\;\;\;\;\;\;\;\;;\;\;z<-\left(a+b\right)\sqrt{3}\sigma_{\psi }\;\;or\;\;z>\left(a+b\right)\sqrt{3}\sigma_{\psi },\\ \left(z+\sqrt{3}\sigma_{\psi }\left(a+b\right)\right)/\left(12ab\sigma_{\psi }^{2}\right)\;\;\;\;;\;\;-\left(a+b\right)\sqrt{3}\sigma_{\psi }\leq z\leq \left(a-b\right)\sqrt{3}\sigma_{\psi },\\ \sqrt{3}/\left(6b\sigma_{\psi }\right)\;\;\;\;\;\;\;\;\;\;\;\;\;;\;\;\left(a-b\right)\sqrt{3}\sigma_{\psi }\leq z\leq \left(b-a\right)\sqrt{3}\sigma_{\psi },\\ \left(-z+\sqrt{3}\sigma_{\psi }\left(a+b\right)\right)/\left(12ab\sigma_{\psi }^{2}\right)\;\;\;;\;\;\left(b-a\right)\sqrt{3}\sigma_{\psi }\leq z\leq \left(a+b\right)\sqrt{3}\sigma_{\psi }, \end{array}\right. \end{array}$$

where $a=\frac{1}{2}\left(\sin\left(\rho_{l,m}\right)\;+\;\sin\left(\frac{\rho_{n,q}}{2}\right)\cos\left(\frac{\rho_{l,m}}{2}\right)\cos\left(\frac{\rho_{l,n}+\rho_{m,q}}{2}\right)\right)$, $b=\frac{1}{2}(\sin\left(\rho_{n,q}\right)+\sin\left(\frac{\rho_{l,m}}{2}\right)\times \cos\left(\frac{\rho_{n,q}}{2}\right)\cos\left(\frac{\rho_{l,n}+\rho_{m,q}}{2}\right))$, and $c=\sin^{2}\left(\frac{\rho_{l,m}}{2}\right)+\sin^{2}\left(\frac{\rho_{n,q}}{2}\right)+\sin\left(\frac{\rho_{l,m}}{2}\right)\sin\left(\frac{\rho_{n,q}}{2}\right)\cos\left(\frac{\rho_{l,n}+\rho_{m,q}}{2}\right)$. Please note that we assume both $\delta_{kl}$ and $\delta_{kn}$ in (33) to be uniformly distributed random variables over $\left[-\sqrt{3}\sigma_{\psi },\;-\sqrt{3}\sigma_{\psi }\right]$. Furthermore, if R/λ is picked large enough so that $4\pi R\sqrt{z+c}/\lambda >3/4$ holds, we have

(34) $$\begin{array}{ccc} \chi_{\psi }\left(\theta ,0\right) & = & \frac{1}{4\pi^{2}}\sqrt{\frac{2\lambda^{3}}{R^{3}}}\int_{z}\frac{\cos\left(\beta \left(\pi \right)\sqrt{z+c}-\frac{3\pi }{4}\right)}{{\left(z+c\right)}^{3/4}}dz\\ & = & \frac{1}{4\pi^{2}}\sqrt{\frac{2\lambda^{3}}{R^{3}}}\int_{z}\frac{2\cos\left(z^{\prime }-\frac{3\pi }{4}\right)}{\sqrt{\beta \left(\pi \right)z^{\prime }}{\left(z+c\right)}^{3/4}}dz^{\prime }. \end{array}$$

Finally, the closed form expression of the above expectation could be easily found using the primitive *G* of $\left(\frac{1}{12ab\sigma_{\psi }^{2}}z^{\prime }+\frac{\sqrt{3}\left(a+b\right)}{12ab\sigma_{\psi }}\right)\cos\left(\frac{4\pi R}{\lambda }\sqrt{z^{\prime }+c}-\frac{3\pi }{4}\right)/{\left(z^{\prime }+c\right)}^{3/4}$ given by

(35) $$\begin{array}{c} G\left(z^{\prime }\right)\;=\;\beta {\left(\pi \right)}^{-5/2}\;\left(\;A^{\prime }\left(\;S\left(\;0.8\sqrt{z^{\prime }}\right)\;-\;C\;\left(0.8\sqrt{z^{\prime }}\right)\right)-\frac{z^{\prime }\left(2z^{\prime }\sin\left(z^{\prime }-\frac{3\pi }{4}\right)+3\cos\left(z^{\prime }-\frac{3\pi }{4}\right)\right)}{12ab\sigma_{\psi }^{2}}\;\;\right), \end{array}$$

where S(x) and C(x) are the Fresnel S and C functions, respectively, and

(36) $$\begin{array}{c} A^{\prime }=\frac{1}{12\sqrt{2}ab\sigma_{\psi }^{2}}\left(5c\beta^{2}\left(\pi \right)+3.8\right)-\frac{5\sqrt{3}\left(a+b\right)}{12\sqrt{2}ab\sigma_{\psi }}\beta^{2}\left(\pi \right). \end{array}$$

As far as $\tau_{\psi }\left(\theta ,0\right)$ is concerned, its closed form expression could also be obtained following similar steps. Indeed, for small $\delta_{kl}$ and large R/λ, we have

(37) $$\begin{array}{c} \tau_{\psi }\left(\theta ,0\right)≃\frac{\lambda^{3}}{8\pi^{4}R^{3}}\left(\int_{-\sqrt{3}\sigma_{\psi }}^{\sqrt{3}\sigma_{\psi }}\frac{\cos\left(\beta \left(\pi \right)\left(\sin\left(\frac{\rho_{l,m}}{2}\right)+\frac{\cos\left(\frac{\rho_{l,m}}{2}\right)}{2}\delta_{k,l}\right)-\frac{3\pi }{4}\right)}{{\left(\sin\left(\frac{\rho_{l,m}}{2}\right)+\frac{\cos\left(\frac{\rho_{l,m}}{2}\right)}{2}\delta_{k,l}\right)}^{0.75}}d\delta_{k,l}\right)\\ \;\;\;\;\;\;\;\times \left(\int_{-\sqrt{3}\sigma_{\psi }}^{\sqrt{3}\sigma_{\psi }}\frac{\cos\left(\beta \left(\pi \right)\left(\sin\left(\frac{\rho_{n,q}}{2}\right)+\frac{\cos\left(\frac{\rho_{n,q}}{2}\right)}{2}\delta_{k,n}\right)-\frac{3\pi }{4}\right)}{{\left(\sin\left(\frac{\rho_{n,q}}{2}\right)+\frac{\cos\left(\frac{\rho_{n,q}}{2}\right)}{2}\delta_{k,n}\right)}^{0.75}}d\delta_{k,n}\right), \end{array}$$

and, hence, it can be solved using the primitive in (35). Please note that the results obtained so far hold for any given angular spread (AS) which is nothing but the variance of the random variables $\theta_{l}$s characterizing the scattering’ strength. However, when AS is relatively small (i.e., low scattering effects), one could derive much more compact and simple expressions both for $\chi_{\psi }\left(\theta ,0\right)$ and $\tau_{\psi }\left(\theta ,0\right)$. In such a case, $\theta_{l}$s are small and so are $\rho_{l,m}$s. Consequently, (29) boils down to

(38) $$\begin{array}{ccc} \chi_{\psi }\left(\theta ,0\right) & ≃ & E_{\delta_{k,l},\delta_{k,n}}\left(\;\frac{2J_{1}\left(2\beta \left(\pi \right)\sin\left(\frac{\rho_{l,m}+\rho_{n,q}+\delta_{k,l}+\delta_{k,n}}{4}\right)\right)}{2\beta \left(\pi \right)\sin\left(\frac{\rho_{l,m}+\rho_{n,q}+2\delta_{\psi_{k}}}{4}\right)}\;\right).\\ & ≃ & E_{\delta_{k,l},\delta_{k,n}}\left(1-\frac{\beta (\pi )}{2}\sin{\left(\frac{\rho_{l,m}+\rho_{n,q}+\delta_{k,l}+\delta_{k,n}}{4}\right)}^{2}\right)\\ & ≃ & 1-\frac{\beta (\pi )}{2}\;\left(\frac{1}{2}+\frac{1}{3\sigma^{2}}\left(\cos\left(\sqrt{3}\sigma_{\psi }\right)-1\right)\right), \end{array}$$

after exploiting in the second line the Taylor series expansion around 0 of the Bessel function. Besides, for small AS, $\tau_{\psi }\left(\theta ,0\right)$ could be rewritten as

(39) $$\begin{array}{ccc} \tau_{\psi }\left(\theta ,0\right) & ≃ & \left(\int_{-\sqrt{3}\sigma }^{\sqrt{3}\sigma }\frac{2J_{1}\left(\rho_{l,m}+\delta_{k,l}\right)}{\rho_{l,m}+\delta_{k,l}}d\delta_{k,l}\right)\left(\int_{-\sqrt{3}\sigma }^{\sqrt{3}\sigma }\frac{2J_{1}\left(\rho_{n,q}+\delta_{k,n}\right)}{\rho_{n,q}+\delta_{k,n}}d\delta_{k,n}\right). \end{array}$$

After performing the variable change $x=\delta_{k,l}/\left(\sqrt{3}\sigma_{\psi }\right)$ and exploiting the equivalence between the Bessel and hypergeometric functions, we obtain

(40) $$\begin{array}{c} \tau_{\psi }\left(\theta ,0\right)≃\left(\;\int_{0}^{1}\;\frac{{}_{2}F_{3}\;\left(2,\frac{3}{2};2,2,3,-12\pi^{2}{\left(\frac{R}{\lambda }\right)}^{2}\rho_{l,m}^{2}\sigma_{\psi }^{2}x\right)}{\sqrt{x}dx}\;\;\right)\left(\;\int_{0}^{1}\;\frac{{}_{2}F_{3}\left(2,\frac{3}{2};2,2,3,-12\pi^{2}{\left(\frac{R}{\lambda }\right)}^{2}\rho_{n,q}^{2}\sigma_{\psi }^{2}x\right)}{\sqrt{x}dx}\;\;\right)\\ \;\;\;\;≃4{}_{3}F_{4}\;\left(\frac{1}{2},2,\frac{3}{2};\frac{3}{2},2,2,3,-12\pi^{2}\;{\left(\frac{R}{\lambda }\right)}^{2}\;\rho_{l,m}^{2}\sigma_{\psi }^{2}\;\right)\;{}_{3}F_{4}\;\left(\frac{1}{2},2,\frac{3}{2};\frac{3}{2},2,2,3,-12\pi^{2}\;{\left(\frac{R}{\lambda }\right)}^{2}\;\rho_{n,q}^{2}\sigma_{\psi }^{2}\;\;\right)\;. \end{array}$$

It follows from (31), (34), (35), (38), and (40) that $w_{\mathrm{PR}}$ depends solely on locally available information at each node, thereby lending itself to a power- and spectrum-efficient RDCB implementation over WSNs even in polychromatic (i.e., scattered) environments. It is worth noting once more, in the absence of localization, DoA, and scatterers estimation errors (i.e., $\sigma_{r}=\sigma_{\psi }=\sigma_{\alpha }=0$), that the proposed P-RDCB, $w_{\mathrm{PR}}$, reduces to the nominal P-DCB solution in given [18] by:

(41) $$\begin{array}{ccc} w_{P} & = & \frac{\Lambda^{-1}h}{K^{2}}{\left(\sum_{l,m,n,q=1}^{L}\alpha_{l}\alpha_{m}^{H}\alpha_{n}^{H}\alpha_{q}\left(\chi (\theta ,0)+(K-1)\tau (\theta ,0)\right)\right)}^{-\frac{1}{2}}. \end{array}$$

where

(42) $$\begin{array}{c} \chi \left(\theta ,0\right)=\frac{2J_{1}\;\;\left(\;\;\;\sqrt{\;\beta \;{\left(\rho_{l,m}\right)}^{2}\;\;+\;\;\beta \;{\left(\rho_{n,q}\right)}^{2}\;\;+\;2\beta \;\left(\rho_{l,m}\right)\;\beta \;\left(\rho_{n,q}\right)\;\cos\left(\frac{\rho_{l,n}+\rho_{m,q}}{2}\right)}\right)}{\;\;\;\sqrt{\;\beta \;{\left(\rho_{l,m}\right)}^{2}\;+\;\beta \;{\left(\rho_{n,q}\right)}^{2}\;+\;2\beta \;\left(\rho_{l,m}\right)\;\beta \;\left(\rho_{n,q}\right)\;\cos\left(\frac{\rho_{l,n}+\rho_{m,q}}{2}\right)}}, \end{array}$$

and

(43) $$\tau \left(\theta ,0\right)=\frac{4J_{1}\left(\beta \left(\rho_{l,m}\right)\right)J_{1}\left(\beta \left(\rho_{n,q}\right)\right)}{\beta \left(\rho_{l,m}\right)\beta \left(\rho_{n,q}\right)}.$$

thereby highlighting yet again both the faced hurdles and the gained advantages of implementing DCB robustness to channel parameter estimation errors.

In what follows, we analyze the performance of the the proposed M-RDCB and P-RDCB solutions.

## 4. Theoretical Performance Analysis of Robustness Gains

In this section, we assess analytically the performance of the new RDCB solutions (i.e., M-RDCB and P-RDCB) in terms of achieved average SNR (ASNR) against the nominal optimal CB solution ${\tilde{w}}_{\mathrm{opt}}$ in (10) so as to assess the theoretical potential gains they could achieve by integrating robustness in their designs. Let $\gamma_{w}=E\left\{P_{w}\left(\phi_{s}\right)/P_{w,n}\right\}$ denote the ASNR achieved by any CB w where the expectation is taken over all node coordinates, forward and backward channels, and channel estimation errors. Unfortunately, the derivation of γ in closed form turns out to be a tedious task, if not impossible. In this work, we propose to study instead another practically appealing metric that is the average signal to average noise ratio (ASANR) ${\bar{\gamma }}_{w}={\bar{P}}_{w}\left(\phi_{s}\right)/{\bar{P}}_{w,n}$ where ${\bar{P}}_{w}\left(\phi_{s}\right)=E\left\{P_{w}\left(\phi_{s}\right)\right\}$ and ${\bar{P}}_{w,n}=E\left\{P_{w,n}\right\}$. Please note that [17,18,19,20,21,22,23] have shown that γ and $\bar{\gamma }$ have approximatively the same behaviors. Let us first derive the average received power ${\bar{P}}_{w}\left(\phi \right)$ from any source located at ϕ using w.

### 4.1. Implementation in Scattering-Free Environments—Option 1

Let us first derive the average beampattern achieved by ${\tilde{w}}_{\mathrm{opt}}$. Exploiting the Taylor series expansion around 0 of the exponential function, we obtain

(44) $$\begin{array}{c} {\bar{P}}_{{\tilde{w}}_{\mathrm{opt}}}\left(\phi \right)\;=\;\frac{1}{K}\;+\;\;\frac{K-1}{K}\;\left[\;\sum_{p=0}^{+\infty }\;\frac{\beta^{p}\left(\phi \right)}{p!}{\left(-j\right)}^{p}E\left(\mu_{k}^{p}\right)E\left(\Delta_{g_{k}}\right)\;\right]\;\;\;\left[\;\sum_{m=0}^{+\infty }\;\frac{\beta^{m}\left(\phi \right)}{m!}{\left(-j\right)}^{m}E\left(\mu_{k}^{m}\right)E\left(\Delta_{g_{k}}\right)\;\right], \end{array}$$

where β(ϕ) = 4π(R/λ)sin(ϕ/2). Besides, we know that

(45) $$J_{n}\left(x\right)=\sum_{p=0}^{+\infty }\frac{{\left(-1\right)}^{p}}{p!(n+p)!}{\left(\frac{x}{2}\right)}^{2p+n},$$

where $J_{n}$ stands for the Bessel functions of first kind. Injecting (45) in (44) leads to

(46) $${\bar{P}}_{{\tilde{w}}_{\mathrm{opt}}}\left(\phi \right)=\frac{1}{K}+\left(1-\frac{1}{K}\right){\left|2\frac{J_{1}\left(\beta \left(\phi \right)\right)}{\beta (\phi )}\right|}^{2}\frac{\sin^{2}\left(\sqrt{3}\sigma_{g}\right)}{3\sigma_{g}^{2}}.$$

It follows from (46) that ${\bar{P}}_{{\tilde{w}}_{\mathrm{opt}}}\left(\phi_{s}=0\right)=\left(1/K\right)+\left(1-\left(1/K\right)\right)\left(\sin^{2}\left(\sqrt{3}\sigma_{g}\right)/\left(3\sigma_{g}^{2}\right)\right)$. Consequently, using the nominal optimal DCB, ${\tilde{w}}_{\mathrm{opt}}$, the power received at Rx decreases with $\sigma_{g}$ due to the channel mismatch. This is not surprising since the design of ${\tilde{w}}_{\mathrm{opt}}$ does not account for such errors. On the other hand, the average beampattern achieved by the proposed M-RDCB can be calculated as

(47) $${\bar{P}}_{w_{{\mathrm{MR}}_{1}}}\left(\phi \right)=\frac{K+K\left(K-1\right)\frac{\sin^{2}\left(\sqrt{3}\sigma_{g}\right)}{3\sigma_{g}^{2}}{\left|2\frac{J_{1}\left(\beta \left(\phi \right)\right)}{\beta (\phi )}\right|}^{2}}{K+K\left(K-1\right)\frac{\sin^{2}\left(\sqrt{3}\sigma_{g}\right)}{3\sigma_{g}^{2}}}.$$

The above result verifies that ${\bar{P}}_{w_{{\mathrm{MR}}_{1}}}\left(\phi_{s}=0\right)=1$ for any given estimation errors. Consequently, the proposed beamformer is much more robust than the nominal optimal DCB.

### 4.2. Implementation in Scattering-Free Environments—Option 2

If Option 2 is adopted, the average beampattern achieved by ${\tilde{w}}_{\mathrm{opt}}$ can be expressed as

(48) $$\begin{array}{ccc} {\bar{P}}_{{\tilde{w}}_{\mathrm{opt}}}\left(\phi \right)\;\;\;\; & = & \;\;\;\;E[\frac{K}{K^{2}}\;+\;\frac{K(K-1)}{K^{2}}\sum_{k=1}^{K}\;\sum_{l=1,l\neq k}^{K}\;\;\;\exp\left(\;\;-j4\pi R\left[\mu_{k}\sin\left(\frac{\phi -\delta_{\psi_{k}}}{2}\right)-\mu_{l}\sin\left(\frac{\phi -\delta_{\psi_{l}}}{2}\right)\right]\right)\\ & & \times \exp\left(j\frac{2\pi }{\lambda }\left(\nu_{k}-\nu_{l}\right)\right)]. \end{array}$$

Let $\xi_{\psi }\left(\phi \right)=E_{\mu_{k},\delta_{\psi_{k}}}\left\{e^{-j4\pi R\mu_{k}\sin\left(\frac{\delta_{\psi_{k}}-\phi }{2}\right)}\right\}\;$. It has already been shown in (22) that

(49) $$\xi_{r}\left(\frac{\pi }{3}\right)={}_{1}\;F_{2}\left(0.5;1,1.5;-3{\left(\pi \frac{\sigma_{r}}{\lambda }\right)}^{2}\right),$$

where ${}_{1}\;F_{2}\left(0.5;1,1.5;x^{2}\right)$ is the the hypergeometric function which has a peak at 0 and decreases when *x* grows large. Besides, we have

(50) $$\begin{array}{c} \xi_{\psi }\left(0\right)≃{}_{1}F_{2}\left(0.5;1.5,2;-3{\left(\pi \frac{R\sigma_{\psi }}{\lambda }\right)}^{2}\right) \end{array}.$$

Injecting (49) and (50) in (48) yields

(51) $${\bar{P}}_{{\tilde{w}}_{\mathrm{opt}}}\left(\phi \right)=\frac{1}{K}+\left(1-\frac{1}{K}\right)\xi_{r}\left(\frac{\pi }{3}\right)\xi_{\psi }\left(\phi \right).$$

It follows from (51) that ${\bar{P}}_{{\tilde{w}}_{\mathrm{opt}}}\left(\phi_{s}=0\right)$ decreases with $\sigma_{r}$ and $\sigma_{\psi }$ due to the channel mismatch. On the other hand, the average beampattern achieved by the proposed M-RDCB, which accounts for such errors, can be determined as

(52) $${\bar{P}}_{w_{{\mathrm{MR}}_{2}}}\left(\phi \right)=\frac{K+K\left(K-1\right)|\xi_{\psi }\left(\phi \right)\xi_{r}\left(\frac{\pi }{3}\right){|}^{2}}{K+K\left(K-1\right)|\xi_{\psi }\left(0\right)\xi_{r}\left(\frac{\pi }{3}\right){|}^{2}}.$$

It follows from (52) that ${\bar{P}}_{w_{{\mathrm{MR}}_{2}}}\left(0\right)=1$ for any localization and DoA estimation errors, in contrast to the nominal optimal DCB, thereby validating again the robustness of the proposed M-RDCB against channel estimation errors. Furthermore, we observe from (51) and (52) that the proposed M-RDCB achieves an important gain over its counterparts in terms of the received desired power, a gain that substantially increases at higher channel estimation errors.

Now, let us turn our attention to the noise powers. Using either ${\tilde{w}}_{\mathrm{opt}}$, $w_{{\mathrm{MR}}_{1}}$, or $w_{{\mathrm{MR}}_{2}}$, the average noise power can be calculated, respectively, as

(53) $$\begin{array}{ccc} {\bar{P}}_{w_{\mathrm{opt}},n} & = & \frac{\sigma_{v}^{2}}{K^{2}}E\left\{\frac{{\left({\tilde{f}}_{k}-\Delta_{f_{k}}\right)}^{H}\left({\tilde{f}}_{k}-\Delta_{f_{k}}\right)}{|{\tilde{f}}_{k}{|}^{2}|}\right\}+\sigma_{n}^{2}\\ & = & \frac{\sigma_{v}^{2}\left(1+\sigma_{f}^{2}\right)}{K^{2}}+\sigma_{n}^{2}, \end{array}$$

and

(54) $$\begin{array}{ccc} {\bar{P}}_{w_{{\mathrm{MR}}_{1}},n}\;\;\; & = & \;\;\;\;\frac{\sigma_{v}^{2}\left(1+\sigma_{f}^{2}\right)}{K\left(1+\sigma_{f}^{2}\right)+K\left(K-1\right)\frac{\sin^{2}\left(\sqrt{3}\sigma_{g}\right)}{3\sigma_{g}^{2}}}\;+\;\sigma_{n}^{2}, \end{array}$$

with Option 1 or

(55) $$\begin{array}{ccc} {\bar{P}}_{w_{{\mathrm{MR}}_{2}},n} & = & \frac{\sigma_{v}^{2}\left(1+\sigma_{f}^{2}\right)}{K\left(1+\sigma_{f}^{2}\right)+K\left(K-1\right)\xi_{\psi }\left(0\right)\xi_{r}\left(\frac{\pi }{3}\right)}\;+\;\sigma_{n}^{2}. \end{array}$$

with Option 2. It could be readily shown from (53)–(55) that ${\bar{P}}_{w_{\mathrm{opt}},n}\geq {\bar{P}}_{w_{{\mathrm{MR}}_{1}},n},\;{\bar{P}}_{w_{{\mathrm{MR}}_{2}},n}$, making ${\bar{\gamma }}_{w_{\mathrm{opt}}}\leq {\bar{\gamma }}_{w_{{\mathrm{MR}}_{1}}},\;{\bar{\gamma }}_{w_{{\mathrm{MR}}_{2}}}$ since ${\bar{P}}_{{\tilde{w}}_{\mathrm{opt}}}\left(\phi_{s}\right)\ll {\bar{P}}_{w_{{\mathrm{MR}}_{1}}}\left(\phi_{s}\right),\;{\bar{P}}_{w_{{\mathrm{MR}}_{2}}}\left(\phi_{s}\right)$.

### 4.3. Implementation in Scattered Environments

From (10) and (25), $P_{{\tilde{w}}_{\mathrm{opt}}}\left(\phi \right)$ turns out to be a complex quotient of several random variables and, hence, deriving the closed form expression of its average is extremely difficult, if not impossible. To circumvent this daunting issue, we propose in this paper to derive instead its asymptotic expression for large *K*. When ${\tilde{w}}_{\mathrm{opt}}$ is implemented in polychromatic environments, the following theorem holds:

**Theorem** **1.** *For large K and any given AS, we have*

(56) $$\begin{array}{ccc} P_{{\tilde{w}}_{\mathrm{opt}}}\left(\phi \right) & ≃ & (\sum_{l,m,n,q=1}^{L}{\tilde{\alpha }}_{l}{\tilde{\alpha }}_{m}^{H}{\tilde{\alpha }}_{n}^{H}{\tilde{\alpha }}_{q}\left(\xi_{r}\left(\rho_{n,l}\right)\chi_{\psi }\left(\theta ,\phi \right)+\left(K-1\right)\xi_{r}\left(\frac{\pi }{3}\right)\tau_{\psi }\left(\theta ,\phi \right)\right)\\ & + & \sum_{\underset{m=q}{l,m,n=1}}^{L}{\tilde{\alpha }}_{l}{\tilde{\alpha }}_{n}^{H}\sigma_{\alpha }^{2}\left(\xi_{r}\left(\rho_{n,l}\right)\chi_{\psi }\left(\theta ,\phi \right)+\left(K-1\right)\xi_{r}\left(\frac{\pi }{3}\right)\tau_{\psi }\left(\theta ,\phi \right)\right))\\ & \times & 4K{\left(\sum_{l=1}^{L}\sum_{m=1}^{L}{\tilde{\alpha }}_{l}^{H}{\tilde{\alpha }}_{m}\xi_{r}\left(\rho_{n,l}\right)\frac{J_{1}\left(\beta \left(\rho_{m,l}\right)\right)}{\beta (\rho_{m,l})}\right)}^{-2}. \end{array}$$

**Proof.**  See Appendix A. □

Since $J_{1}\;\left(x\right)/x$ has a maximum at 0 and decreases rapidly with *x*, we have $J_{1}\;\left(\beta \left(\rho_{m,l}\right)\;\right)\;/\;\beta \left(\rho_{m,l}\right)\gg \chi_{\psi }\left(\theta ,0\right)$ and, hence, $P_{{\tilde{w}}_{\mathrm{opt}}}\left(0\right)<1$. This means that the nominal optimal DCB is unable to satisfy the constraint in (5) due to channel estimation errors. Now, the next theorem introduces the asymptotic expression for $P_{w_{\mathrm{PR}}}\left(\phi \right)$.

**Theorem** **2.** *For large K and any given AS, we have*

(57) $$\begin{array}{ccc} P_{w_{\mathrm{PR}}}\left(\phi \right) & ≃ & (\sum_{l,m,n,q=1}^{L}{\tilde{\alpha }}_{l}{\tilde{\alpha }}_{m}^{H}{\tilde{\alpha }}_{n}^{H}{\tilde{\alpha }}_{q}\left(\xi_{r}\left(\rho_{n,l}\right)\chi_{\psi }\left(\theta ,\phi \right)+\left(K-1\right)\xi_{r}\left(\frac{\pi }{3}\right)\tau_{\psi }\left(\theta ,\phi \right)\right)\\ & + & \sum_{\underset{m=q}{l,m,n=1}}^{L}{\tilde{\alpha }}_{l}{\tilde{\alpha }}_{n}^{H}\sigma_{\alpha }^{2}\left(\xi_{r}\left(\rho_{n,l}\right)\chi_{\psi }\left(\theta ,\phi \right)+\left(K-1\right)\xi_{r}\left(\frac{\pi }{3}\right)\tau_{\psi }\left(\theta ,\phi \right)\right))\\ & \times & (\sum_{l,m,n,q=1}^{L}{\tilde{\alpha }}_{l}{\tilde{\alpha }}_{m}^{H}{\tilde{\alpha }}_{n}^{H}{\tilde{\alpha }}_{q}\left(\xi_{r}\left(\rho_{n,l}\right)\chi_{\psi }\left(\theta ,0\right)+\left(K-1\right)\xi_{r}\left(\frac{\pi }{3}\right)\tau_{\psi }\left(\theta ,0\right)\right)\\ & + & \sum_{\underset{m=q}{l,m,n=1}}^{L}{\tilde{\alpha }}_{l}{\tilde{\alpha }}_{n}^{H}\sigma_{\alpha }^{2}\left(\xi_{r}\left(\rho_{n,l}\right)\chi_{\psi }\left(\theta ,0\right)+\left(K-1\right)\xi_{r}\left(\frac{\pi }{3}\right)\tau_{\psi }\left(\theta ,0\right)\right)){}^{-1}. \end{array}$$

**Proof.**  See Appendix A. □

It follows from (57) that $P_{w_{\mathrm{PR}}}\left(0\right)=1$ when *K* is large enough. Therefore, the power received from the source *S* is substantially improved if $w_{\mathrm{PR}}$ is implemented in lieu of ${\tilde{w}}_{\mathrm{opt}}$. Furthermore, we have

(58) $$\begin{array}{ccc} \lim_{K\to \infty }\frac{P_{w_{RP},n}}{P_{{\tilde{w}}_{\mathrm{opt}},n}} & = & \;\;\frac{{\left(\lim_{K\to \infty }\left(1/K\right){\tilde{h}}^{H}{\tilde{\Lambda }}^{-1}\tilde{h}\right)}^{2}}{{\left(\lim_{K\to \infty }\left(1/K\right){\tilde{h}}^{H}{\tilde{\Lambda }}^{-1}h\right)}^{2}}\\ & = & \;\;\;{\left(\frac{2\sum_{l,m=1}^{L}{\tilde{\alpha }}_{l}^{H}{\tilde{\alpha }}_{m}\xi_{r}\left(\rho_{m,l}\right)\frac{J_{1}\left(\beta \left(\rho_{m,l}\right)\right)}{\beta (\rho_{m,l})}}{\xi_{r}\left(\frac{\pi }{3}\right)\left(\sum_{l,m,n,q=1}^{L}{\tilde{\alpha }}_{l}{\tilde{\alpha }}_{m}^{H}{\tilde{\alpha }}_{n}^{H}{\tilde{\alpha }}_{q}\tau_{\psi }\left(\theta ,0\right)+\sum_{\underset{m=q}{l,m,n=1}}^{L}\;\;{\tilde{\alpha }}_{l}{\tilde{\alpha }}_{n}^{H}\sigma_{\alpha }^{2}\tau_{\psi }\left(\theta ,0\right)\right)}\right)}^{2}\\ & \gg & \;\;{\left(\frac{\sum_{l,m=1}^{L}{\tilde{\alpha }}_{l}^{H}{\tilde{\alpha }}_{m}\xi_{r}\left(\rho_{m,l}\right)\frac{J_{1}\left(\beta \left(\rho_{m,l}\right)\right)}{\beta (\rho_{m,l})}}{\xi_{r}\left(\frac{\pi }{3}\right)\left(\sum_{\underset{m=q}{l,m,n=1}}^{L}{\tilde{\alpha }}_{l}{\tilde{\alpha }}_{n}^{H}\tau_{\psi }\left(\theta ,0\right)\right)\left(\sigma_{\alpha }^{2}+{\left|{\tilde{\alpha }}_{m}\right|}^{2}\right)}\right)}^{2}. \end{array}$$

Since $J_{1}\left(\beta \left(\rho_{m,l}\right)\right)/\beta \left(\rho_{m,l}\right)\gg \tau_{\psi }\left(\theta ,0\right)$ and both $\sigma_{\alpha }^{2}$ and ${\left|{\tilde{\alpha }}_{m}\right|}^{2}$ are lower than 1, $\left(P_{w_{\mathrm{PR}},n}/P_{{\tilde{w}}_{\mathrm{opt}},n}\right)>1$ holds when *K* is large enough. Consequently, in such a condition, ${\bar{\gamma }}_{w_{\mathrm{PR}}}>{\bar{\gamma }}_{{\tilde{w}}_{\mathrm{opt}}}$, thereby proving the superiority of the new P-RDCB against the nominal optimal DCB in polychromatic environments.

## 5. Numerical Evaluation Results

This section evaluates numerically the performance of the proposed M-RDCB and P-RDCB techniques and gauge them against the nominal optimal CB solution ${\tilde{w}}_{\mathrm{opt}}$ in (10) and to the RDCB in [32] to emphasize on one hand the need for and the benefits of implementing robustness in DCB, and to assess on the other hand the performance gains of the proposed solutions against the best representative RDCB benchmark. The empirical quantities are obtained by averaging over $10^{5}$ random realizations of of $r_{k}$, $\psi_{k}$, ${\left[f\right]}_{k}$ for k=1,…,K and $\alpha_{l}$, $\theta_{l}$ for l=1,…,L. In all simulations, we assume that the number of rays or chromatics is L=6, R/λ=1, and the noises’ powers $\sigma_{n}^{2}$ and $\sigma_{v}^{2}$ are 10 dB below the source transmit power.

**Figure 2:**
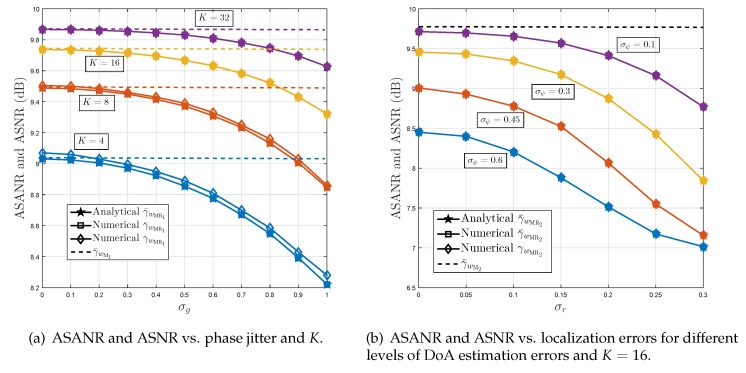
Average signal to average noise ratio (ASANR) and average signal to noise ratio (ASNR) of proposed monochromatic robust distributed collaborative beamforming (M-RDCB) in monochromatic environments under: (**a**) implementation Option 1, and (**b**) implementation Option 2.

Figure 2 plots the ASANR and ASNR achieved by the proposed beamformer in monochromatic (i.e., scattering-free) environments versus the variances of channel errors $\sigma^{2}=\sigma_{g}^{2}=\sigma_{f}^{2}$, $\sigma_{r}^{2}$, and $\sigma_{\psi }^{2}$ for different values of *K*. Figure 2a considers implementation Option 1 which results in a phase jitter while Figure 2b considers implementation Option 2 which results in localization and DoA estimation errors. We observe from these figures that the analytical and empirical curves of ${\bar{\gamma }}_{w_{{\mathrm{MR}}_{1}}}$ and ${\bar{\gamma }}_{w_{{\mathrm{MR}}_{2}}}$ match perfectly thereby validating the correctness of the derivations in Section 4. Furthermore, we notice that the ASNR and ASANR curves remain very close for K≤8 or coincide almost perfectly otherwise, thereby proving the insightfulness of the ASANR metric. As far as the proposed M-RDCB performance is concerned, it is able to achieve optimal performance, even for small *K*, when the channel estimation errors are relatively small to moderate (i.e., $\sigma_{g}\leq 1$ in Option 1 or $\sigma_{r}\leq 0.2$ and $\sigma_{\psi }\leq 0.1$ in Option 2). This confirms the robustness of the new M-RDCB. For extremely large errors, however, it looses only a fraction of a dB. Actually, with the advances made during the two last decades in the field of phase synchronization, localization, and DoA estimation, these channel estimation errors are often very small, making our beamformer’s performance optimal if advanced parameter estimation algorithms are adopted. Nevertheless, the latter naturally come with increased complexity and cost, which certainly burden those of WSN nodes. In this context, the proposed M-RDCB offers the possibility of using inaccurate but low-cost estimation algorithms at negligible performance losses, making it a more appealing and practical for cost-effective WSN deployment in real-world conditions. All these observations corroborate all the results of Section 3.

**Figure 3:**
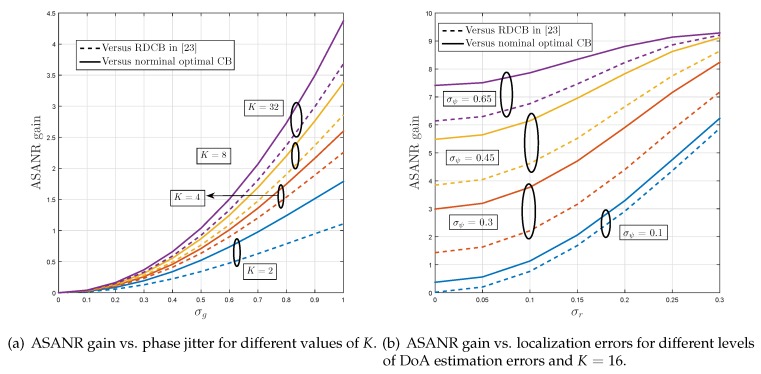
ASANR gain of proposed M-RDCB in monochromatic environments under: (**a**) implementation Option 1, and (**b**) implementation Option 2.

Figure 3 displays the achieved ASANR gain of proposed M-RDCB against the nominal optimal DCB and the M-RDCB in [32] for different values of *K*. Figure 3a considers the first implementation option while Figure 3b considers the second. We observe from these figures that M-RDCB largely outperforms both benchmarks for any given *K*, $\sigma_{\psi }$, $\sigma_{r}$, and $\sigma_{\alpha }$. For instance, when Option 1 is adopted, it achieves for K=32 ASANR gains of 4.3 and 3.7 dB against the nominal optimal DCB and RDCB in [32], respectively. If Option 2 is adopted, these ASANR gains increase, respectively, to as much as 9.4 and 9.2 dB when $\sigma_{\psi }=0.65$ and $\sigma_{r}=0.3$. As could be observed from Figure 3, these gains increase rapidly with both *K* and channel estimation errors. These observations corroborate all the results of Section 4 and verify the net superiority of robust M-RDCB techniques in scattering-free environments.

**Figure 4:**
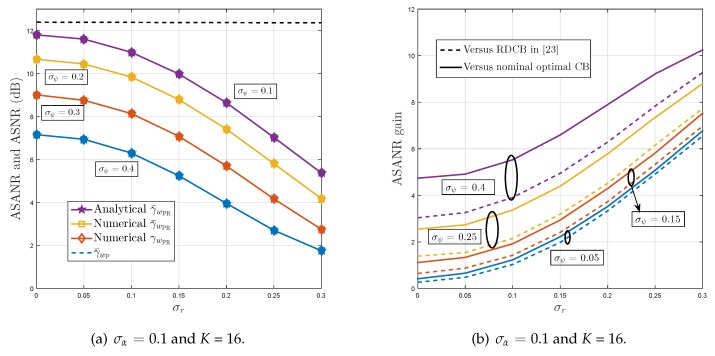

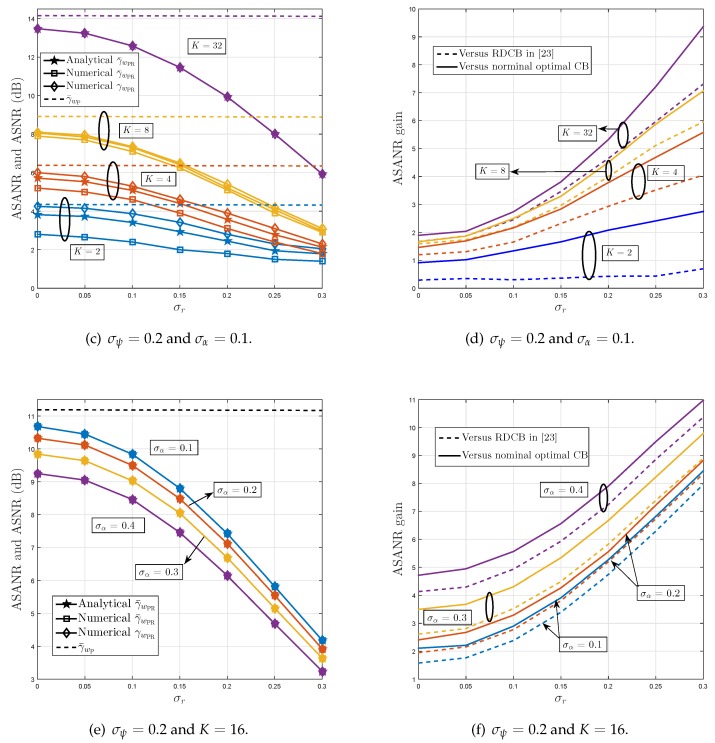
ASANR, ASNR, and ASANR gain of proposed P-RDCB in polychromatic environments.

Figure 4 shows the ASANR, ASNR, and the ASANR gain of the proposed P-RDCB in polychromatic (i.e., scattered) environments. Figure 4a,c,e plot its achieved ASANR and ASNR versus *K*, $\delta_{r}$, $\delta_{\psi }$, and $\sigma_{\alpha }$ while Figure 4b,d,f compare its achieved ASANR with those of the nominal optimal DCB and RDCB in [32]. As expected, $w_{\mathrm{PR}}$ approaches the optimal ASANR performance level even in polychromatic environments, and that is for all tested AS values. In such environments, $w_{\mathrm{PR}}$ achieves ASANR gains of until 10.4 and 9.4 dB against both benchmarks, respectively. As can be observed from Figure 4b,d,f these gains increase rapidly with both *K* and channel estimation errors. For instance, according to Figure 4f, the ASANR gain over the two benchmarks increases by approximatively 225% against both when $\sigma_{r}^{2}$ is twice as large or 73.5% and 68%, respectively, when α is four times as large. These additional observations further verify the high robustness of the proposed P-RDCB against channel estimation errors, key practical feature that allows its cost-effective integration in real-world WSN applications. Again, according to Figure 4, we verify that the analytical and empirical values of ${\bar{\gamma }}_{w_{\mathrm{PR}}}$ match perfectly when K>8 or are very close otherwise. All these observations corroborate once more the discussion of Section 4.

## 6. Conclusions

We have proposed a new DCB solution robust against major channel estimation impairments, namely, phase synchronization, localization, direction-of-arrival (DoA), and/or channel scatterers estimation errors over dual-hop transmissions from a source to a destination communicating through a WSN of *K* nodes. Exploiting an efficient asymptotic approximation at large *K*, we have developed alternative RCB solutions that not only account for estimation errors, but also adapt to different implementation scenarios and wireless propagation environments ranging from monochromatic to polychromatic ones. Besides, in contrast to existing techniques, our new RCB solutions are distributed in that they do not require any information exchange among nodes, thereby dramatically improving both WSN spectral and power efficiencies. Simulation results have confirmed that the proposed RDCB techniques are much more robust in terms of achieved SNR against channel estimation errors than best representative CB benchmarks, yet at much lower complexity, power cost, and overhead, making them suitable for WSN deployment in the harsh environments that characterize operation in real-world conditions.

## Appendix A

Let

(A1) $$g_{k}\left(\phi \right)=e^{-j\frac{2\pi }{\lambda }r_{k}\cos\left(\phi -\psi_{k}\right)},$$

denote the channel from a source located at ϕ to the *k*-th node. From the definition of ${\tilde{w}}_{\mathrm{opt}}$ in (10),

(A2) $$\begin{array}{ccc} \lim_{K\to \infty }P_{{\tilde{w}}_{\mathrm{opt}}}\left(\phi \right) & = & \lim_{K\to \infty }\frac{{\left({\tilde{h}}^{H}{\tilde{\Lambda }}^{-1}h\left(\phi \right)\right)}^{H}\left({\tilde{h}}^{H}{\tilde{\Lambda }}^{-1}h\left(\phi \right)\right)}{{\left({\tilde{h}}^{H}{\tilde{\Lambda }}^{-1}\tilde{h}\right)}^{2}}\\ & = & \frac{\sum_{l,m,n,q=1}^{L}{\tilde{\alpha }}_{l}\alpha_{m}^{H}{\tilde{\alpha }}_{n}^{H}\alpha_{q}\left(\lim_{K\to \infty }\left(1/K\right)\Pi_{1}+\lim_{K\to \infty }\left(1/K\right)\Pi_{2}\right)}{\lim_{K\to \infty }{\left({\tilde{h}}^{H}{\tilde{\Lambda }}^{-1}{\tilde{h}}^{H}\right)}^{2}}, \end{array}$$

where h(ϕ)=f⊙g(ϕ),

(A3) $$\begin{array}{ccc} \Pi_{1} & = & \sum_{k=1}^{K}e^{-j\frac{r_{k}}{R}\left(\beta \left(\rho_{l,m}+\delta_{k,l}-\phi \right)\sin\left(x-\psi_{k}\right)+\beta \left(\rho_{n,q}+\delta_{k,n}-\phi \right)\sin\left(y-\psi_{k}\right)\right)}e^{-j\frac{2\pi }{\lambda }\left(\vartheta_{k,n}-\vartheta_{k,l}\right)}, \end{array}$$

and

(A4) $$\begin{array}{c} \Pi_{2}=\sum_{k=1}^{K}e^{-j\frac{r_{k}}{R}\left(\beta \left(\rho_{l,m}+\delta_{k,l}-\phi \right)\sin(x-\psi_{k}\right)}e^{-j\frac{2\pi }{\lambda }\vartheta_{k,l}}\sum_{p\neq k}^{K}e^{-j\frac{r_{k}}{R}\left(\beta \left(\rho_{n,q}+\delta_{k,n}-\phi \right)\sin(y-\psi_{p}\right)}e^{-j\frac{2\pi }{\lambda }\vartheta_{p,n}}. \end{array}$$

Exploiting the fact that $r_{k}$ and $\psi_{k}$ are mutually independent, we have

(A5) $$\begin{array}{ccc} \lim_{K\to \infty }\frac{1}{K}\Pi_{1} & \;\;=\;\; & \;\;\;\;\lim_{K\to +\infty }\frac{1}{K}\sum_{k=1}^{K}e^{-j\frac{r_{k}}{R}\left(\beta \left(\rho_{l,m}+\delta_{k,l}-\phi \right)\sin\left(x-\psi_{k}\right)+\beta \left(\rho_{n,q}+\delta_{l,n}-\phi \right)\sin\left(y-\psi_{k}\right)\right)}e^{-j\frac{2\pi }{\lambda }\left(\vartheta_{k,n}-\vartheta_{k,l}\right)}\\ & = & \;\;\;\;KE_{r_{k},\psi_{k}}\;\;\left(\;e^{-j\frac{r_{k}}{R}\left(\beta \left(\rho_{l,m}+\delta_{k,l}-\phi \right)\sin\left(x-\psi_{k}\right)+\beta \left(\rho_{n,q}+\delta_{k,n}-\phi \right)\sin\left(y-\psi_{k}\right)\right)}\;\right)\;E_{\vartheta_{k}}\;\;\;\left(\;e^{-j\frac{2\pi }{\lambda }\left(\vartheta_{k,n}-\vartheta_{k,l}\right)}\;\right)\\ & = & \;\;\;\;K\chi_{\psi }\left(\theta ,\phi \right)\xi_{r}\left(\rho_{n,l}\right). \end{array}$$

Furthermore, we have

(A6) $$\begin{array}{c} \lim_{K\to \infty }\frac{1}{K}\Pi_{2}=\lim_{K\to +\infty }\;\frac{1}{K}\;\sum_{k=1}^{K}\;e^{-j\frac{r_{k}}{R}\left(\beta \left(\rho_{l,m}+\delta_{k,l}-\phi \right)\sin(x-\psi_{k}\right)}\sum_{p\neq k}^{K}\;e^{-j\frac{r_{k}}{R}\left(\beta \left(\rho_{n,q}+\delta_{k,n}-\phi \right)\sin(y-\psi_{p}\right)}e^{-j\frac{2\pi }{\lambda }\left(\vartheta_{p,n}+\vartheta_{k,l}\right)}\\ =K\left(K-1\right)E_{\delta_{\psi }}\;\;\left(\;\frac{4J_{1}\left(\beta \left(\rho_{l,m}-\phi +\delta_{k,l}\right)\right)\;J_{1}\left(\beta \left(\rho_{n,q}-\phi +\delta_{k,n}\right)\right)}{\beta \left(\rho_{l,m}-\phi +\delta_{k,l}\right)\beta \left(\rho_{n,q}-\phi +\delta_{k,n}\right)}\;\right)E_{\vartheta_{p}}\;\;\left(e^{-j\frac{2\pi }{\lambda }\left(\vartheta_{p,n}\right)}\right)\;\;E_{\vartheta_{k}}\;\;\left(e^{-j\frac{2\pi }{\lambda }\left(\vartheta_{k,l}\right)}\right)\\ =K\left(K-1\right)\tau_{\psi }\left(\theta ,\phi \right)\xi_{r}^{2}\left(\frac{\pi }{3}\right). \end{array}$$

Let us now turn our attention to the denominator of the right hand side of (A2). Using the law of large numbers, one could obtain

(A7) $$\begin{array}{ccc} \lim_{K\to \infty }{\left({\tilde{h}}^{H}{\tilde{\Lambda }}^{-1}\tilde{h}\right)}^{H}\left({\tilde{h}}^{H}{\tilde{\Lambda }}^{-1}\tilde{h}\right) & = & \;\;\;\;\lim_{K\to +\infty }\;\frac{1}{K}{\left(\sum_{l,m=1}^{L}\sum_{k=1}^{K}{\tilde{\alpha }}_{l}^{*}\alpha_{m}e^{-j\beta \left(\rho_{m,l}+\delta_{kl}\right)\kappa_{klm}}e^{-j\frac{2\pi }{\lambda }\vartheta_{kl}}\right)}^{2}\\ & = & \;\;\;\;K^{2}\;\;{\left(\sum_{l,m=1}^{L}\sum_{k=1}^{K}{\tilde{\alpha }}_{l}^{*}\alpha_{m}E_{\delta_{\psi }}\;\;\left(e^{-j\beta \left(\rho_{m,l}+\delta_{kl}\right)\kappa_{klm}}\right)\;\;E_{\vartheta_{k}}\;\;\left(e^{-j\frac{2\pi }{\lambda }\left(\vartheta_{k,l}\right)}\right)\;\;\;\right)}^{2}\\ & ≃ & \;\;\;4K^{2}{\left(\sum_{l=1}^{L}\sum_{m=1}^{L}{\tilde{\alpha }}_{l}^{H}{\tilde{\alpha }}_{m}\xi_{r}\left(\rho_{n,l}\right)\frac{J_{1}\left(\beta \left(\rho_{m,l}\right)\right)}{\beta (\rho_{m,l})}\right)}^{2}. \end{array}$$

Substituting (A5)–(A7) in (A2) yields to (56). As far as $\lim_{K\to \infty }P_{w_{p}}$ is concerned, we have

(A8) $$\begin{array}{ccc} \lim_{K\to \infty }P_{w_{\mathrm{PR}}}\left(\phi \right) & = & \lim_{K\to \infty }\frac{{\left({\tilde{h}}^{H}{\tilde{\Lambda }}^{-1}h\left(\phi \right)\right)}^{H}\left({\tilde{h}}^{H}{\tilde{\Lambda }}^{-1}h\left(\phi \right)\right)}{{\left({\tilde{h}}^{H}{\tilde{\Lambda }}^{-1}h\right)}^{2}}\\ & = & \frac{\lim_{K\to \infty }\left(1/K\right)\Pi_{1}+\lim_{K\to \infty }\left(1/K\right)\Pi_{2}}{\lim_{K\to \infty }{\left({\tilde{h}}^{H}{\tilde{\Lambda }}^{-1}h^{H}\right)}^{2}}. \end{array}$$

Besides, we have

(A9) $$\begin{array}{c} \lim_{K\to \infty }{\left({\tilde{h}}^{H}{\tilde{\Lambda }}^{-1}h\right)}^{H}\left({\tilde{h}}^{H}{\tilde{\Lambda }}^{-1}h\right)=K\sum_{l,m,n,q=1}^{L}{\tilde{\alpha }}_{l}\alpha_{m}^{H}{\tilde{\alpha }}_{n}^{H}\alpha_{q}(E_{\vartheta_{k}}\left(e^{-j\frac{2\pi }{\lambda }\left(\vartheta_{k,n}-\vartheta_{k,l}\right)}\right)\\ \times E_{r_{k},\psi_{k}}\;\;\left(e^{-j\frac{r_{k}}{R}\left(\beta \left(\rho_{l,m}+\delta_{k,l}\right)\sin\left(x-\psi_{k}\right)+\beta \left(\rho_{n,q}+\delta_{k,n}\right)\sin\left(y-\psi_{k}\right)\right)}\right)\\ +E_{\delta_{\psi }}\;\;\left(\;\frac{4J_{1}\left(\beta \left(\rho_{l,m}-\phi +\delta_{k,l}\right)\right)\;J_{1}\left(\beta \left(\rho_{n,q}-\phi +\delta_{k,n}\right)\right)}{\beta \left(\rho_{l,m}-\phi +\delta_{k,l}\right)\beta \left(\rho_{n,q}-\phi +\delta_{k,n}\right)}\;\right)E_{\vartheta_{k}}\;\;\left(e^{-j\frac{2\pi }{\lambda }\left(\vartheta_{p,n}\right)}\right)E_{\vartheta_{k}}\;\;\left(e^{-j\frac{2\pi }{\lambda }\left(\vartheta_{k,l}\right)}\right))\\ ≃K\;\;\;\sum_{l,m,n,q=1}^{L}\;\;\;\;{\tilde{\alpha }}_{l}\alpha_{m}^{H}{\tilde{\alpha }}_{n}^{H}\alpha_{q}\left(\xi_{r}\left(\rho_{n,l}\right)\chi_{\psi }\left(\theta ,0\right)+\left(K-1\right)\xi_{r}^{2}\left(\frac{\pi }{3}\right)\tau_{\psi }\left(\theta ,0\right)\right)\\ =K\;\;\;\sum_{l,m,n,q=1}^{L}\;\;\;\;{\tilde{\alpha }}_{l}{\tilde{\alpha }}_{m}^{H}{\tilde{\alpha }}_{n}^{H}{\tilde{\alpha }}_{q}\left(\xi_{r}\left(\rho_{n,l}\right)\chi_{\psi }\left(\theta ,0\right)+\left(K-1\right)\xi_{r}^{2}\left(\frac{\pi }{3}\right)\tau_{\psi }\left(\theta ,0\right)\right)\\ +K\;\;\;\sum_{\underset{m=q}{l,m,n=1}}^{L}\;\;\;\;{\tilde{\alpha }}_{l}{\tilde{\alpha }}_{n}^{H}\sigma_{\alpha }^{2}\left(\xi_{r}\left(\rho_{n,l}\right)\chi_{\psi }\left(\theta ,0\right)+\left(K-1\right)\xi_{r}^{2}\left(\frac{\pi }{3}\right)\tau_{\psi }\left(\theta ,0\right)\right)\;\;. \end{array}$$

Finally, $\lim_{K\to \infty }P_{w_{\mathrm{PR}}}\left(\phi \right)$ could be easily obtained using (A5)–(A9).

## References

[1] Felici-Castell S., Navarro E.A., Pérez-Solano J.J., Segura-García J., García-Pineda M. (2017). Practical considerations in the implementation of collaborative beamforming on wireless sensor networks. Sensors.

[2] Ochiai H., Mitran P., Poor H.V., Tarokh V. (2005). Collaborative beamforming for distributed wireless ad hoc sensor networks. IEEE Trans. Sig. Proc..

[3] Bao X., Liang H., Han L. (2018). Transmission optimization of social and physical sensor nodes via collaborative beamforming in cyber-physical-social systems. Sensors.

[4] Zaidi S., Affes S., Vilaipornsawai U., Zhang L., Zhu P. Wireless access virtualization strategies for future user-centric 5G networks. Proceedings of the IEEE GC Workshops 2016.

[5] Zaidi S., Smida O.B., Affes S., Vilaipornsawai U., Zhang L., Zhu P. QoS-Based virtualization of user equipment in 5G networks. Proceedings of the IEEE IWCMC 2018.

[6] Wang H., Yao Z., Yang J., Fan Z. (2018). A novel beamforming algorithm for GNSS receivers with dual-polarized sensitive arrays in the joint space–time-polarization domain. Sensors.

[7] Jayaprakasam S., Rahim K.A., Leow C.Y. (2017). Distributed and collaborative beamforming in wireless sensor networks: classifications, trends, and research directions. IEEE Commun. Surv. Tutor..

[8] Zarifi K., Affes S., Ghrayeb A. (2010). Collaborative null-steering beamforming for uniformly distributed wireless sensor networks. IEEE Trans. Sig. Proc..

[9] Liu X., Jia Y., Wen Z., Zou J., Li S. (2018). Beamforming Design for Full-Duplex SWIPT with Co-Channel Interference in Wireless Sensor Systems. Sensors.

[10] Liu H.X., Wen Z., Liu D., Zou J., Li S. (2019). Joint source and relay beamforming design in wireless multi-hop sensor networks with SWIPT. Sensors.

[11] Bengtsson M., Ottersten B. (2000). Low-complexity estimators for distributed sources. IEEE Trans. Sig. Proc..

[12] Huang J., Wang P., Wan Q. (2012). Collaborative beamforming for wireless sensor networks with arbitrary distributed sensors. IEEE Commun. Lett..

[13] Zarifi K., Ghrayeb A., Affes S. (2010). Distributed beamforming for wireless sensor networks with improved graph connectivity and energy efficiency. IEEE Trans. Sig. Proc..

[14] Han Z., Poor H.V. (2007). Lifetime improvement in wireless sensor networks via collaborative beamforming and cooperative transmission. IET Microwav. Antennas Propagat..

[15] Dong L., Petropulu A.P., Poor H.V. (2008). A cross-layer approach to collaborative beamforming for wireless ad hoc networks. IEEE Trans. Sig. Proc..

[16] Zarifi K., Zaidi S., Affes S. (2011). A distributed amplify-and-forward beamforming technique in wireless sensor networks. IEEE Trans. Sig. Proc..

[17] Zaidi S., Affes S. (2014). Distributed collaborative beamforming in the presence of angular scattering. IEEE Trans. Commun..

[18] Zaidi S., Affes S. (2015). Distributed collaborative beamforming design for maximized throughput in interfered and scattered environments. IEEE Trans. Commun..

[19] Zaidi S., Affes S. (2012). SNR and throughput analysis of distributed collaborative beamforming in locally-scattered environments. Wirel. Commun. Mob. Comp..

[20] Zaidi S., Affes S., Kandil N. (2015). Accurate range-free localization in multi-hop wireless sensor networks. IEEE Trans. Commun..

[21] El Assaf A., Zaidi S., Affes S., Kandil N. (2016). Robust ANNs-based WSN localization in the presence of anisotropic signal attenuation. IEEE Wirel. Commun. Lett..

[22] Zaidi S., Ben Smida O., Affes S., Valaee S. Distributed zero-forcing AF beamforming for energy-efficient communications in networked smart cities. Proceedings of the IEEE PIMRC 2017.

[23] Zaidi S., Hmidet B., Affes S. (2015). Power-constrained distributed implementation of SNR-optimal collaborative beamforming in highly-scattered environments. IEEE Wirel. Commun. Lett..

[24] Asztely D., Ottersten B. The effects of local scattering on direction of arrival estimation with MUSIC. Proceedings of the IEEE ICASSP 1998.

[25] Zheng G., Wong K.K., Paulraj A., Ottersten B. (2009). Robust collaborative-relay beamforming. IEEE Trans. Sig. Proc..

[26] Chen P., Yang Y., Wang Y., Ma Y. (2018). Robust Adaptive Beamforming with Sensor Position Errors Using Weighted Subspace Fitting-Based Covariance Matrix Reconstruction. Sensors.

[27] Mahboobi B., Soleimani-Nasab E., Ardebilipour M. (2014). Outage probability based robust distributed beam-forming in multi-user cooperative networks with imperfect CSI. Wirel. Person. Commun..

[28] Ben Smida O., Zaidi S., Affes S., Valaee S. Low-cost robust distributed collaborative beamforming against implementation impairments. Proceedings of the IEEE GLOBECOM 2018.

[29] Huang X., Wu H.C., Principe J.C. (2007). Robust blind beamforming algorithm using joint multiple matrix diagonalization. IEEE Sens. J..

[30] Liu F., Du R., Wu J., Zhou Q., Zhang Z., Cheng J. (2018). Multiple Constrained *ℓ*_2_-Norm Minimization Algorithm for Adaptive Beamforming. IEEE Sens. J..

[31] Ponukumati D., Gao F., Xing C. (2013). Robust peer-to-peer relay beamforming: A probabilistic approach. IEEE Commun. Lett..

[32] Sadr M.A.M., Mahboobi B., Mehrizi S., Attari M.A., Ardebilipour M. (2016). Stochastic robust collaborative beamforming: non-regenerative relay. IEEE Trans. Commun..

[33] Tsinos C.G., Vlachos E., Berberidis K. Distributed blind adaptive computation of beamforming weights for relay networks; In Proceedings of the IEEE PIMRC 2013, London, UK, 8–11 September 2013.

[34] Li J., Petropulu A.P., Poor H.V. (2011). Cooperative transmission for relay networks based on second-order statistics of channel state information. IEEE Trans. Sig. Proc..

[35] Nassab V.H., Shahbazpanahi S., Grami A., Luo Z.Q. (2008). Distributed beamforming for relay networks based on second-order statistics of the channel state information. IEEE Trans. Sig. Proc..

[36] Gong S., Wu S.X., Man-Cho So A., Huang X. (2017). Distributionally robust collaborative beamforming in D2D relay networks with interference constraints. IEEE Trans. Wirel. Commun..

[37] Sadr M.A.M., Attari M.A., Amiri R. (2018). Robust relay beamforming against jamming attack. IEEE Commun. Lett..

[38] Chalise B.K., Vandendorpe L. (2009). Optimization of MIMO relays for multipoint-to-multipoint communications: nonrobust and robust designs. IEEE Trans. Sig. Proc..

[39] Chalise B.K., Vandendorpe L. (2010). MIMO relay design for multipoint-to-multipoint communications with imperfect channel state information. IEEE Trans. Sig. Proc..

[40] Van Veen B.D., Buckley K.M. (1988). Beamforming: A versatile approach to spatial filtering. IEEE ASSP Mag..

[41] Affes S., Gazor S., Grenier Y. (1996). An algorithm for multisource beamforming and multitarget tracking. IEEE Trans. Signal Process..

[42] Thibault I., Corazza G.E., Deambrogio L. Phase synchronization algorithms for distributed beamforming with time varying channels in wireless sensor networks. Proceedings of the IEEE IWCMC 2011.

[43] Shi S., Zhu S., Gu X., Hu R. Extendable carrier synchronization for distributed beamforming in wireless sensor networks. Proceedings of the IEEE IWCMC 2016.

[44] Ming W. Distributed node location algorithm using non-anchor node clustering. Proceedings of the IEEE ICCSE 2016.

[45] Sriploy P., Uthansakul P., Uthansakul M. An effect of imperfection in node location estimation on distributed beamforming. Proceedings of the IEEE ECTI-CON 2012.

